# Integrated Lactylome Characterization Reveals the Molecular Dynamics of Protein Regulation in Gastrointestinal Cancers

**DOI:** 10.1002/advs.202400227

**Published:** 2024-07-17

**Authors:** Yangmiao Duan, Hanxiang Zhan, Qin Wang, Bohao Li, Huiru Gao, Duanrui Liu, Qinchen Xu, Xin Gao, Zhenya Liu, Peng Gao, Guangwei Wei, Yunshan Wang

**Affiliations:** ^1^ Key Laboratory for Experimental Teratology of the Ministry of Education Department of Cell Biology School of Basic Medical Sciences Cheeloo College of Medicine Shandong University Jinan Shandong 250012 China; ^2^ Division of Pancreatic Surgery Department of General Surgery Qilu Hospital Shandong University Jinan Shandong 250012 China; ^3^ Department of Anesthesiology Qilu Hospital Shandong University Jinan Shandong 250012 China; ^4^ Department of Clinical Laboratory The Second Hospital Cheeloo College of Medicine Shandong University Jinan Shandong 250033 China; ^5^ Department of Clinical Laboratory Shandong Provincial Hospital Affiliated to Shandong First Medical University Jinan Shandong 250021 China; ^6^ Key Laboratory for Experimental Teratology of the Ministry of Education and Department of Pathology School of Basic Medical Sciences Cheeloo College of Medicine Shandong University Jinan 250012 China

**Keywords:** CBX3, epigenetic rewiring, gastrointestinal cancer, lactylome, pan‐cancer pattern

## Abstract

Lysine lactylation (Kla) plays a vital role in several physiological processes. However, the cancer‐specific modulation of Kla in gastrointestinal (GI) tumors requires systematic elucidation. Here, global lactylome profiling of cancerous and adjacent tissues is conducted from 40 patients with GI cancer and identified 11698 Kla sites. Lactylome integration revealed that Kla affects proteins involved in hallmark cancer processes, including epigenetic rewiring, metabolic perturbations, and genome instability. Moreover, the study revealed pan‐cancer patterns of Kla alterations, among which 37 Kla sites are consistently upregulated in all four GI cancers and are involved in gene regulation. It is further verified that lactylation of CBX3 at K10 mediates its interaction of CBX3 with the epigenetic marker H3K9me3 and facilitates GI cancer progression. Overall, this study provides an invaluable resource for understanding the lactylome landscape in GI cancers, which may provide new paths for drug discovery for these devastating diseases.

## Graphical abstract

In this study, a workflow is adapted to systematically identify lactylation modifications at the proteomic scale in cancerous and adjacent tissues from 40 patients with GI cancer. Integrated lactylome analysis reveals the connections and alterations in key functional pathways affected by dysregulated Kla modifications in GI cancer, which may provide new paths for drug discovery for these devastating diseases.

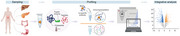

## 1 Introduction

Gastrointestinal (GI) cancers are a heterogeneous group of malignancies representing more than 26% of cancer incidence and 35% of all cancer‐related mortalities.^[^ ^1^, ^2^ ^]^ Recent studies on the proteogenomic landscapes of individual human GI cancers have revealed extensive molecular mechanisms of the tumor progression.^[^ ^3^, ^4^, ^5^, ^6^ ^]^ However, the limited targeted therapies for GI cancers underscore the urgent need to develop effective molecular targets for the diagnosis and treatment of this disease.

Metabolic reprogramming is a hallmark of cancer, in which tumor cells rely mainly on glycolysis for energy supply and metabolic intermediates to support tumor growth.^[^ ^7^, ^8^, ^9^ ^]^ Lactate, which is generated during aerobic glycolysis, accumulates excessively in tumor cells. With the development of high‐resolution liquid chromatography coupled with tandem mass spectrometry (LC‐MS/MS),^[^ ^10^, ^11^, ^12^ ^]^ various acylation modifications using intermediate metabolites as substrates have been identified. In 2019, lactate‐derived lysine lactylation (Kla) in histone proteins was identified as a new type of posttranslational modifications (PTMs).^[^ ^13^ ^]^ Accumulating evidence suggests that protein lactylation is involved in diverse biological processes such as tumor proliferation,^[^ ^14^ ^]^ nervous system regulation,^[^ ^15^ ^]^ and metabolic regulation.^[^ ^16^ ^]^ Given the enhanced glycolysis and lactate overproduction in cancer cells, the discovery of lactylation provides a new perspective on the important mechanisms of lactate function in cancer progression.

Here, we adapted a workflow to systematically identify all Kla modifications at the proteomic scale in malignant and adjacent tissues of patients in a prospectively collected GI cancer cohort, including liver, pancreatic, colorectal, and gastric cancer samples. More than 11 000 Kla sites on 3156 key proteins were identified, and the molecular characteristics of lactylation modifications across distinct GI tumors were also elucidated. Integrated lactylome analysis further revealed that the connections and alterations in key functional pathways are affected by dysregulated Kla modifications in GI cancer, including chromobox 3 (CBX3), a member of the heterochromatin protein family that plays a vital role in gene transcriptional regulation.^[^ ^17^ ^]^ Notably, CBX3 lactylation at lysine 10 (CBX3 K10la) was confirmed to be dramatically upregulated in GI cancers, and functional analysis revealed that CBX3 K10la is instrumental in regulating gene expression and the malignancy of GI tumors.

## 2 Results

### 2.1 Widespread Lysine Lactylation Modifications Mapped to the GI Cancer Proteome

**Figure 1:**
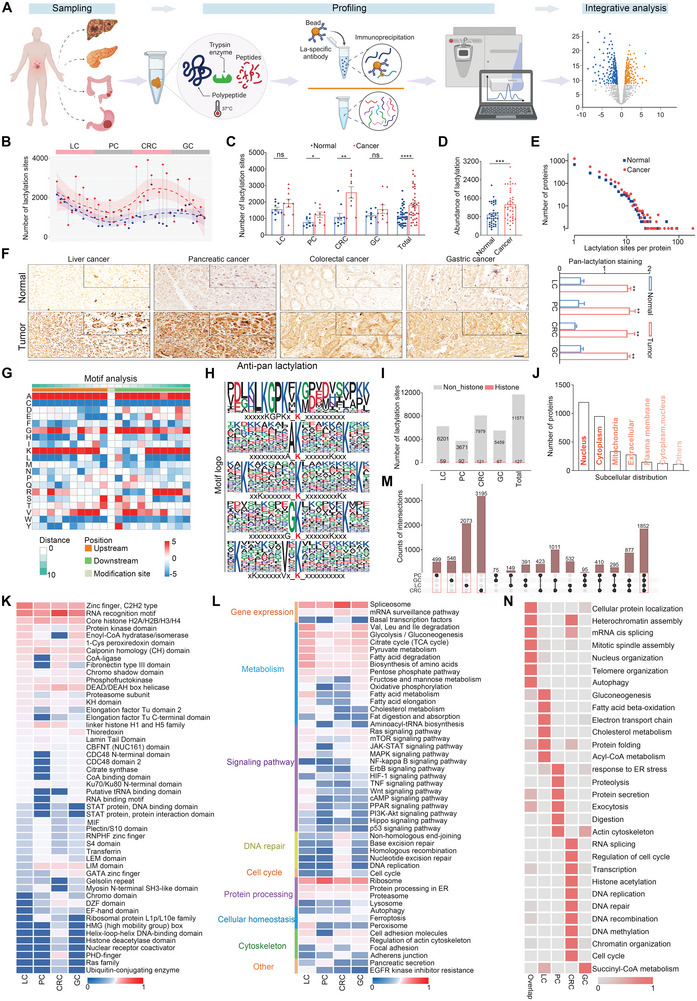
Widespread lysine lactylation modifications mapped to the GI cancer proteome. A) Schematic of the lysine lactylome and proteome analyses of four types of human GI tumors (liver cancer, n = 10; pancreatic cancer, n = 10; colorectal cancer, n = 10; gastric cancer, n = 10) and adjacent tissues by label‐free quantitation. The image was created with BioRender. B) Overview of the Kla identifications in GI tumor (red, n = 40) and non‐tumor (blue, n = 40) samples. The samples are arranged based on the GI cancer subtypes. The pairwise samples are annotated with grey straight lines. The shading that underlies the lasso curves denotes the 95% confidence intervals. C) Box plots of the Kla site identification in GI samples (LC, n = 10; PC, n = 10; CRC, n = 10; GC, n = 10; and Total, n = 40). D) Quantitative analysis of the overall abundance of Kla in GI samples (n = 40 samples per group). E) Numbers of Kla sites per protein in GI tumors and NATs (n = 40 samples per group). F) IHC staining showing the pan‐Kla level in GI cancer and NATs. The graph shows the quantitative analysis of pan‐lactylation levels in the four types of GI cancers (n = 3 for each type of cancer). The data of negative control assay with IgG antibody was shown in Figure S3C (Supporting Information). Scale bars denote 100 µm, and 50 µm (the inserts). G) Heatmap of the 21 amino‐acid compositions of the Kla site showing the frequency of the different amino acids in specific positions flanking the lactylated lysine. Different colors represent the preference of each residue in the position of a 21 amino‐acid‐long sequence context (red indicates greater possibility, whereas blue refers to less possibility). H) The ten amino acids up‐ and downstream of the Kla using Motif‐X are analyzed and the top five motifs with the highest scores are shown. The height of each letter corresponds to the frequency of that amino‐acid residue in that position. The central K refers to the lactylated lysine. I) Numbers of detected Kla sites on non‐histone and histone proteins. J) Subcellular location of all lactylated proteins identified. K,L) Protein domain (K) and KEGG pathway (L) analyses of the enrichment of lactylated proteins in the four GI cohorts using the DAVID database. Note that 0 and 1 in the legend represents the normalized *p* values, with larger values indicating more significant enrichment. M) Intersection of all Kla sites identified from four GI cohorts. N) Gene Ontology biological process analyses of the lactylated proteins identified from single cancer or all four GI samples. Data are presented as mean ± SEM (C, D, and F). *p* values were calculated by two‐tailed Welch's t test (C, D, and F). *p < 0.05, **p < 0.01, ***p < 0.001 and ****p < 0.0001. ns indicates non‐significant. See also Figures S1–S3 (Supporting Information).

To obtain a comprehensive molecular understanding of Kla in GI tumor progression, paired tumor and normal adjacent tissues (NATs) from 40 patients with liver, pancreatic, colorectal, or gastric cancer (*n* = 10 for each type of cancer) were selected for lactylome analysis based on stringent criteria (**Figure** **1**A; Figure S1A, Supporting Information; Table S1, Supporting Information). Global lactylome and proteome analyses were performed using a robust label‐free quantification approach and on the same MS system with consistent quality control (Figure S1B‐D, Supporting Information). We identified 9690 proteins from 40 GI tumors and NATs, as well as 11 698 Kla sites over 3156 proteins, among which 6260 Kla sites were in liver, 3763 in pancreatic, 8100 in colorectal, and 5526 in gastric tissues (Figures S1E and S2A‐H, Supporting Information; Table S2, Supporting Information). A case‐by‐case review showed that the number of Kla sites and proteins identified in the tumors was significantly higher than those identified in paired non‐tumor tissues (Figure 1B,C; Figure S2I,J, Supporting Information). The abundance and types of lactylation modifications were globally increased in GI cancers compared to NAT tissues, and aggressive tumors were identified with more Kla modifications (Figure 1D,E; Figure S2K,L, Supporting Information). Consistently, immunohistochemical (IHC) staining of different GI tumors and NAT tissues with pan‐lactylation antibodies revealed a significantly higher signal intensity in cancer tissues (Figure 1F; Figure S3A‐C, Supporting Information), suggesting that the GI proteome is widely targeted by Kla modifications in cancer tissues.

To investigate the amino acid sequence preference for lactylation modification in human GI tissues, Kla motif analysis of all 11 616 lactylated peptides was performed using Motif‐X. IceLogo heat maps were used to assess the preference of each residue surrounding the Kla sites in a 21 amino‐acid‐long sequence context against the human GI proteome (Figure 1G). Alanine and lysine residues occurred both upstream and downstream, and had the highest probability of occurrence in lactylated peptides. Cysteine, leucine, and tryptophan residues had the lowest probability of occurrence in lactylated peptides. The five most conserved motifs were alanine, lysine, valine, and glycine, which surround the acceptor lactylated lysine. Analysis of lactylated lysine sites indicated a strong bias for alanine (Figure 1H). In addition, a comparative analysis of conserved motifs based on Kla showed slight differences between tumors and NATs, and some were only found in the tumor or NAT samples (Figure S3D,E, Supporting Information).

We collected the Kla sites on non‐histones detected in this study, and among the 11 698 Kla sites detected in this study, 11 571 sites were found on non‐histone protein, indicating that Kla is a prevalent modification beyond histone proteins (Figure 1I). Subcellular localization analysis of lactylated proteins revealed a widespread cellular impact, especially in the nucleus and cytosol, hinting at the profound biological functions of Kla through the modification of nuclear and cytosolic proteins (Figure 1J). Next, protein domain and pathway enrichment analyses were performed to describe the potential function of Kla, which showed a significant enrichment of functionally conserved domains and biological processes, including gene regulation, metabolism, genome stability, and other hallmark cancer pathways (Figure 1K,L; Figure S3F, Supporting Information).

To search for pan‐GI tissue patterns of Kla modification, the overlap among lactylation sites identified from distinct GI samples was analyzed, and 1852 Kla sites were found to occurr across all four cohorts. Notably, the occurrence of lysine lactylation modifications also showed specific patterns in each GI sample, with 2073 only identified in the liver, 499 in pancreatic, 3195 in colorectal, and 546 in gastric samples (Figure 1M). The frequencies of these specific Kla sites in each tissue type were analyzed and ranked (Figure S3G, Supporting Information). Detailed Gene Ontology analysis using the DAVID database indicated that these common or specific lactylproteins in distinct GI cancers were enriched in different functional biological processes, which may reflect their role in specific regulation at the tissue level, beyond that of protein expression alone (Figure 1N). Collectively, our data indicate that widespread Kla modifications span proteins with a broad functional distribution in the four types of GI cancer, implying the potential regulation of Kla in GI cancer pathogenesis.

### 2.2 Integrated Lactylome Analysis of Kla Alterations in GI Cancer and their Clinical Relevance

**Figure 2:**
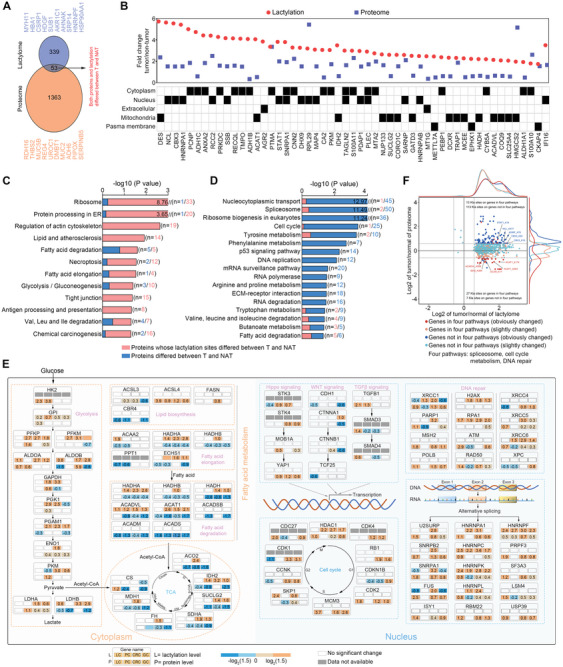
Integrated lactylome analysis of Kla alterations in GI cancer. A) Overlap of proteins containing differential Kla sites (lactylome) and DEPs (proteome). B) Fold changes between tumors and matched NATs for the 53 GI cancer‐associated proteins and their subcellular localization (n = 40 samples per group). C,D) Pathway enrichment analysis based on proteins containing differential Kla sites (C) or differentially expressed proteins (D) in GI tumors by DAVID database. *p* values were calculated by two‐sided Wilcoxon rank‐sum test. E) Overview of cancer hallmark pathways based on integrated lactylome analysis. Protein and Kla changes of single tumor indicated in comparison with non‐tumor GI tissues. F) Quadrant chart depicting the alteration of Kla sites in tumors compared with NATs. Every point represents one Kla site. Labels of “obviously” or “slightly” represent the absolute value of log1.5 of (tumor/adjacent ratio) > 0.66 or ≤ 0.66 in either proteome or lactylome. Four pairs of number located in each corner represent the total number of obviously changed points (gene in four pathways listed or not in four pathways) in each quadrant. The displayed Kla sites all satisfied adjusted *p* < 0.05 in comparing tumors (n  =  40) and adjacent tissues (n  =  40). *p* values were calculated by two‐sided Wilcoxon rank‐sum test (E, F). See also Figure S4 (Supporting Information).

Among the 11 698 Kla sites identified in the four GI sample cohorts, 1345 were quantified. In essence, 850 Kla sites spanning 393 proteins showed a significantly increased abundance in GI cancers (adjusted *p* < 0.05) (Figure S4A,B, Supporting Information; Table S3A, Supporting Information). In addition, among the 6001 proteins quantified in at least 50% of the paired samples, 1292 were upregulated and 124 were downregulated by >1.5‐fold (adjusted *p* < 0.05) (Figure S4C,D, Supporting Information; Table S3B, Supporting Information), suggesting that there may exist a potential connection between these differentially expressed proteins (DEPs) and GI cancer development. Moreover, dysregulated Kla sites and DEPs were differentially distributed across cellular compartments, implying compartment‐specific regulation of differential Kla or DEP in GI cancer (Figure S4E,F, Supporting Information). Overlap analysis of the lactylome and proteome datasets demonstrated that 53 proteins were significantly dysregulated in the two datasets (**Figure** **2**A). These were defined as GI cancer‐associated proteins, most of which had greater changes in lactylsite abundance than in the corresponding protein abundance. Cellular compartment analysis confirmed that 20 of the 53 GI cancer‐associated proteins were localized in the cytoplasm, 16 in the nucleus, 15 in the mitochondrial, two in the extracellular, and three in the plasma (Figure 2B). KEGG pathway analysis indicated that proteins with differential lactylation sites were distributed across numerous functional pathways, particularly metabolic processes, including fatty acid metabolism, glycolysis/gluconeogenesis, and amino acid metabolism (Figure 2C). In addition to several metabolic pathways, DEPs implicated in the spliceosome and cell‐cycle/DNA repair processes were specifically enriched in GI tumors (Figure 2D). Subtype‐specific pathway enrichment analysis clearly demonstrated the molecular features of the four GI cancer types, further assessing the functional impact of differential Kla sites across distinct GI tumors. The significantly altered proteins with lactylation in the representative pathways of the four types of cancer have roles in hallmark pathways, including the spliceosome, glucose metabolism, fatty acid metabolism, cell cycle, and DNA repair (Figure 2E; Figure S4G, Supporting Information). Therefore, both the lactylome and proteome analyses were in agreement with the biological significance of gene regulation, metabolic reprogramming, and genome instability in GI cancer. Although tumor development is accompanied by abnormal protein expression, the fold changes in the lactylation levels of most Kla sites were higher than those of the corresponding proteins in GI cancers (Figure S4H, Supporting Information). We further divided all Kla sites into four quadrants according to their tumor‐to‐NAT ratios and corresponding protein abundance, followed by pathway enrichment analysis. Apparently, the number of Kla sites in the two right quadrants (Kla‐upregulation irrespective of protein) was greater than that in the left quadrants (Kla‐downregulation irrespective of protein), owing to higher lactate levels in the tumors. Most differential Kla sites on proteins enriched in the spliceosome, metabolism, and cell‐cycle/DNA repair pathways were slightly altered (Figure 2F). These results further indicate the specific regulatory role of the high‐lactate environment in cancer hallmark pathways.

**Figure 3:**
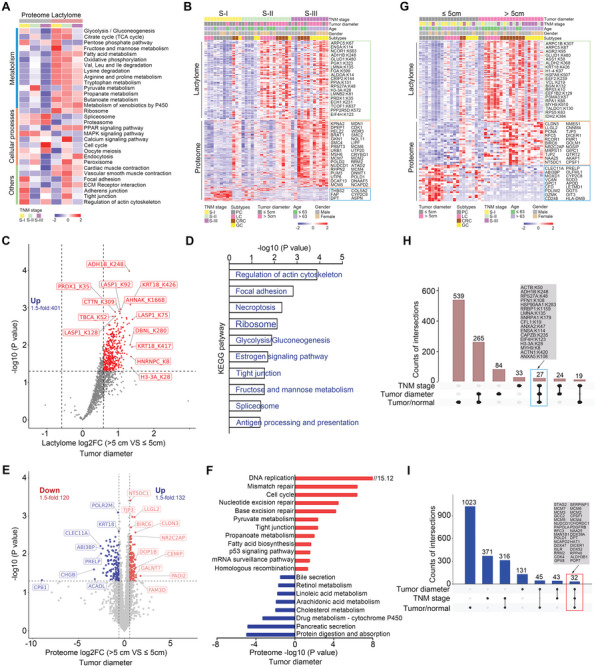
Pathway alterations and differential expression of the lactylome according to clinical traits. A) Heat map of alteration pathways in different tumor stages. Left, alteration pathways identified from the proteome; right, alteration pathways identified from the lactylome. The color of each cell represents the average ssGSEA enrichment scores of that subtype; red denotes activation and blue denotes inhibition. Blank cells represent non‐enrichment (S‐I, n = 12; S‐II, n = 16; and S‐III, n = 12). B) Hierarchical clustering of Kla sites or proteins correlated with different tumor stages. C) Volcano plots depicting differential Kla sites in comparison to tumor diameters. Red and blue dots indicate upregulated and downregulated Kla sites in the indicated tumor diameter (≤5 cm, n = 19; >5 cm, n = 21). D) Bar plots showing the enriched pathways of differential Kla sites from Figure 3C. No pathways are shown for proteins harboring downregulated Kla sites. E) Volcano plots depicting DEPs in comparison of tumor sizes across patients. Red and blue dots indicate upregulated and downregulated proteins in indicated tumor diameter (≤5 cm, n = 19; >5 cm, n = 21). F) Bar plots depicting the enriched pathways of DEPs from Figure 3E. Upregulated and downregulated pathways are indicated in red (right) and blue (left) bars. G) Hierarchical clustering of differential Kla sites or DEPs (adjusted *p* < 0.01) in comparison of indicated clinicopathological characteristics. H,I) Overlap of differential Kla sites (H) or DEPs (I) related to the indicated clinicopathological characteristics. *p* values were calculated by two‐sided Wilcoxon rank‐sum test (D, F, and G).

GI cancers with a poor prognosis exhibit aggressive clinicopathological characteristics, such as a higher tumor stage and larger tumor diameter. Therefore, determining the signatures of Kla or proteins associated with these aggressive clinicopathological characteristics is clinically significant. Gene set enrichment analysis identified 31 core pathways with signatures associated with metabolism and gene regulation enriched in stage II and III GI cancers, and proteins involved in cell adhesion and extracellular matrix organization enriched in stage I cancers (**Figure** **3**A). In addition, 103 Kla sites and 762 proteins that could distinguish histopathologically aggressive tumors from less aggressive tumors were identified by performing supervised comparisons (Figure 3B; Table S4A,B, Supporting Information). We further showed that 401 differential Kla sites that regulate metabolic reprogramming, spliceosome, and focal adhesion were enriched in larger tumors with diameter > 5 cm (Figure 3C,D; Table S5A, Supporting Information). From the proteomic data, 252 DEPs involved in multiple functional pathways were also identified in larger tumors (Figure 3E,F; Table S5B, Supporting Information). More stringent unsupervised hierarchical clustering of these differential Kla sites or DEPs (adjusted *p* < 0.01) almost entirely separated larger tumors from tumors with diameter ≤ 5 cm (Figure 3G). Moreover, 27 Kla sites and 32 proteins were found not only differentially expressed in GI tumors, but also correlated with both tumor stage and diameter (Figure 3H,I), suggesting that these signatures may be of clinical significance for general GI cancer progression.

### 2.3 Regulation of Histone Lactylation

**Figure 4:**
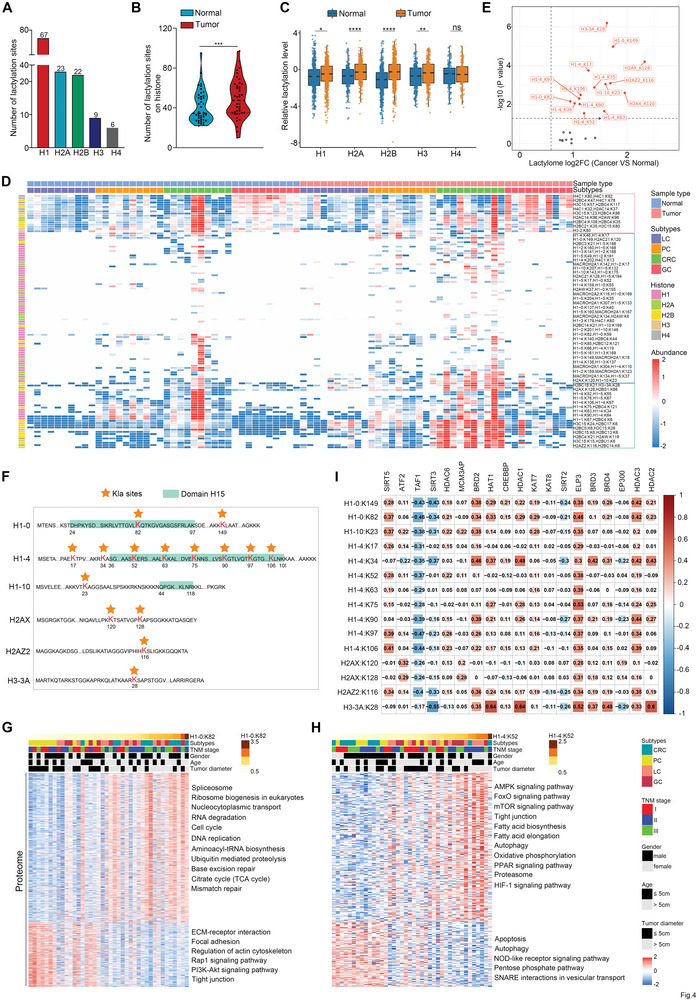
Regulation of histone lactylation. A) Number of detected Kla sites on different histones in GI samples (n = 80). B) Histogram shows the numbers of detected histone Kla sites in GI tumor or NATs (n = 40 samples per group). C) Lactylation‐level changes in specific histone between GI tumors and NATs are shown (n = 40 samples per group). D,E) Heatmap and volcano plot analyses indicating Kla sites altered on different histones in GI cancer. Fifteen red dots based on volcano plot analysis (E) indicate upregulated Kla sites in GI cancer (n = 40 samples per group). F) Analysis of differential Kla sites in the amino acid sequences of histone molecules. G,H) Associations of the indicated Kla abundance with clinicopathological characteristics and proteome profiling. I) Associations between histone acetylases and deacetylases and histone lactylation levels. Data are presented as mean ± SEM (B, C). *p* values were calculated by two‐tailed Welch's t test (B, C) and two‐sided Wilcoxon rank‐sum test (I). *p < 0.05, **p < 0.01, ***p < 0.001 and ****p < 0.0001. ns indicates non‐significant.

Histones with Kla have been confirmed to be critical for gene expression regulation.^[^ ^18^ ^]^ A total of 127 Kla sites spanned distinct histones in our study (**Figure** **4**A), and the number of Kla sites identified in the histones of GI tumors was significantly higher than that in normal tissues (Figure 4B). We further demonstrated that the lactylation levels of different histones increased globally in GI cancer (Figure 4C). Unsupervised hierarchical clustering of the 127 Kla sites in histones separated normal tissues from GI cancers (Figure 4D), and 15 differential Kla sites were found in the GI cancer cohort (Figure 4E). Moreover, seven out of the 15 differential Kla sites were located in the histone H15 domain, which is an essential component of the chromatin structure (Figure 4F). Through elucidating the correlation between the lactylation levels of these differential Kla sites and other proteomic data, we found that tumors with higher lactylation of H1‐0: K82 or H1‐4: K52 showed proteomic dysregulation of multiple functional pathways (Figure 4G,H).

However, the mechanism underlying histone lactylation remains poorly understood. The enzymes that are related to histone acetylation modifications have been confirmed to regulate various other acylation modifications.^[^ ^19^, ^20^ ^]^ We also found positive associations between the expression levels of BRD2, a member of the bromodomain and extra‐terminal domain (BET) family,^[^ ^21^ ^]^ and ELP3 protein and histone lactylation abundance, as well as negative associations between SIRT3 protein levels and most differential Kla sites, indicating that these enzymes may regulate histone Kla levels. In addition, BRD2 partially prevented delactylation by decreasing the accessibility of the erasers to Kla (Figure 4I). Collectively, our data indicate that the histones are widely and significantly targeted by lactylation modifications in GI cancer, which may play an important role in chromatin remodeling and gene transcription.

### 2.4 Comparisons of Tumor‐NAT Revealed Subtype‐Specific Signatures across Distinct GI Tumors

**Figure 5:**
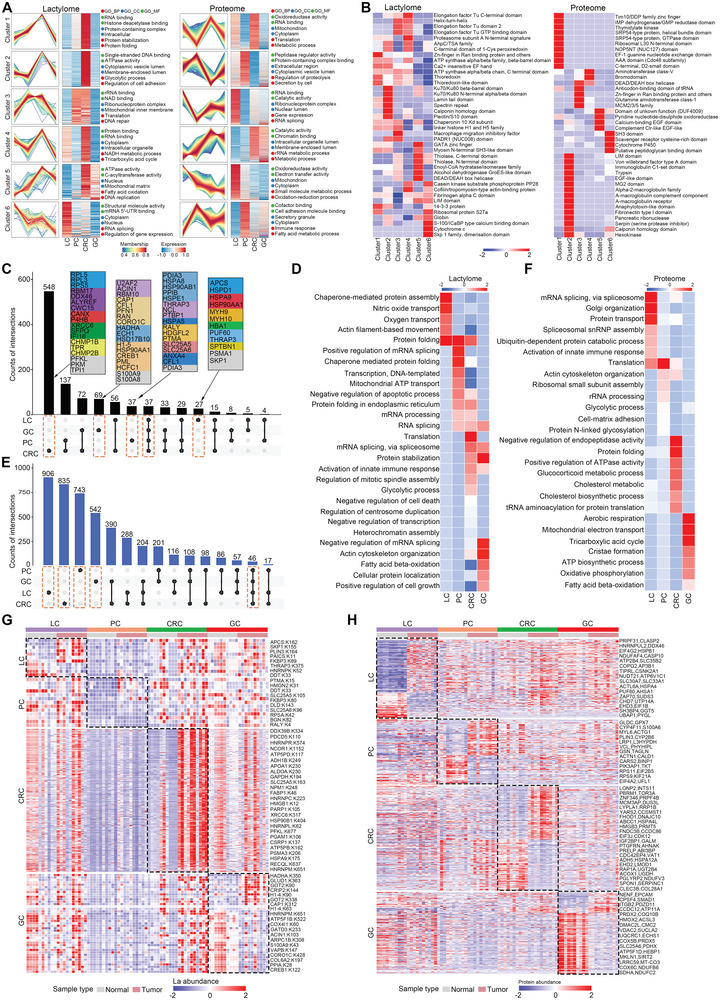
Characterizing the subtype‐specific signatures across distinct GI tumors. A) Pan‐cancer patterns of Kla (left) or protein (right) regulation across distinct GI tumors (LC, n = 10; PC, n = 10; CRC, n = 10; and GC, n = 10). B) Protein domain enrichment analysis based on the lactylated sites or proteins in each pattern cluster obtained from Figure 5A. C,E) Intersection of differential Kla sites (C) or DEPs (E) identified from the four GI cohorts. D,F) Gene Ontology biological process analysis of the subtype‐specific differential Kla sites (D) or DEPs (F) identified from the four GI cohorts. G,H) Hierarchical clustering of the subtype‐specific Kla sites (G) or proteins (H) in each GI cohort (adjusted *p* < 0.01).

GI cancers are heterogeneous diseases at both the cellular and histological levels, and the molecular dynamics of protein regulation across distinct GI cancers need to be elucidated. To elucidate the pan‐cancer patterns of protein regulation across distinct types of GI cancers, clustering analysis was performed based on alterations in proteins or Kla sites using the fuzzy c‐means algorithm, and six patterns were displayed. Gene Ontology and protein domain enrichment analyses were further performed for these patterns (**Figure** **5**A, B). Cluster 1 based on the lactylome highlighted lactylated proteins with a Zn‐finger, 14‐3‐3 protein, and a thioredoxin domain involved in molecular function of nucleic acid binding. Clusters 2, 4, and 5 based on the lactylome, and Clusters 1, 4, 5, and 6 based on the proteome data specifically enriched in proteins involved in metabolism, including glycolysis, the tricarboxylic acid (TCA) cycle, and fatty acid metabolism, indicating that the genes in these clusters may be critical for the regulation of GI cancer metabolic reprogramming. Cluster 6 based on the lactylome and Cluster 3 based on the proteome data highly expressed genes with LIM domain, Zn‐finger, and DEAD/DEAH box helicase domains involved in RNA splicing and gene expression processes. These results further highlight the importance of lactylome integration in identifying the dysregulation of molecular homeostasis during tumor progression.

To investigate the differential Kla sites related to specific tumor subtypes, we analyzed the intersections of differential Kla sites identified in the four GI cancers, and found 37 Kla sites that were differentially expressed across all four cohorts. Importantly, the differential Kla sites showed a more discrete pattern in the distinct subtypes, with 27 sites identified only in liver cancer, 37 in pancreatic cancer, 548 in colorectal cancer, and 69 in gastric cancer (Figure 5C; Table S6A, Supporting Information). Detailed Gene Ontology analysis based on these subtype‐specific Kla sites was further explored (Figure 5D), and the results implied that different types of GI tumors were driven by distinct molecular pathways in the context of protein lactylation, thus providing unique diagnostic or therapeutic opportunities. In addition, the intersections of DEPs identified in the four GI cancers were also analyzed, and 46 DEPs were found across all four cohorts. Moreover, 906 genes were only differentially expressed in liver cancer, 743 in pancreatic cancer, 835 in colorectal cancer, and 542 in gastric cancer (Figure 5E; Table S6B, Supporting Information). Biological process enrichment based on these subtype‐specific DEPs revealed distinctive features among distinct GI cancers (Figure 5F). More stringent unsupervised hierarchical clustering of subtype‐specific Kla sites or DEPs (adjusted *p* < 0.01) (Figure 5G,H) almost entirely separated normal tissues from GI cancers, suggesting that these signatures may be relevant to tumorigenesis in GI cancers.

### 2.5 Dysregulated Kla Sites are Widely Related to Gene Regulation in GI Cancers

**Figure 6:**
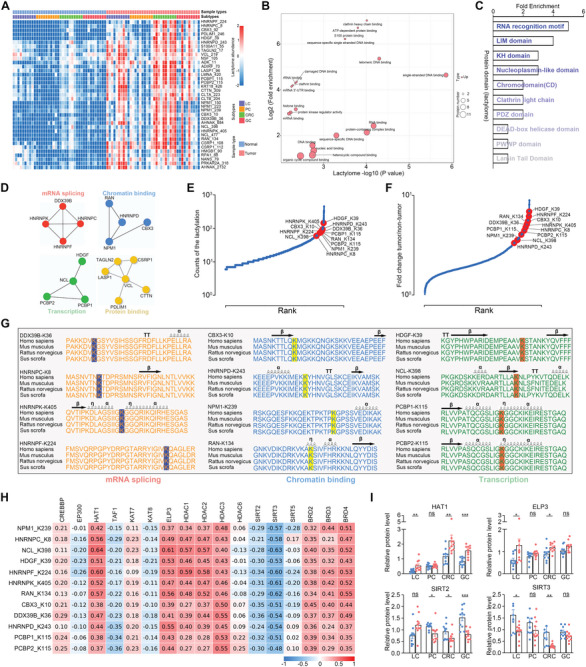
Protein lysine lactylation widely related to gene regulation in GI cancers. A) Hierarchical clustering of the common differential Kla sites identified in all four GI tumors. B) Biological process analysis of the proteins with common differential Kla sites from four GI cohorts. X axes were plotted with ‐log10 (Fisher's exact test p‐value) and represent the enrichment significance. Y axes were plotted with log2 (Fold enrichment) and represented the enrichment degree. The node size in the graph represents the number of proteins in the specified term. C,D) Protein domain and complex enrichment analysis based on common differential lactylproteins from Figure 6A. E) Relative frequencies of Kla sites ranked against all proteins in the dataset. The twelve Kla sites related to gene regulation are highlighted. F) Fold changes of Kla level in GI cancer ranked against all proteins in the dataset. G) Conservation of the twelve Kla sites from different species is shown. H) Associations between acetylases and deacetylases and the indicated lactylation sites. I) Boxplots for the quantification of the indicated proteins in GI tumor and NATs (LC, n = 10; PC, n = 10; CRC, n = 10; GC, n = 10). Data are presented as mean ± SEM (I). *p* values were calculated by two‐sided Wilcoxon rank‐sum test (I). *p < 0.05, **p < 0.01, ***p < 0.001, and ****p < 0.0001. ns indicates non‐significant. See also Figures S5 and S6 (Supporting Information).

Next, we focused on the common differential Kla sites across the four GI tumors. Unsupervised clustering revealed that the levels of 37 Kla sites were consistently upregulated in GI cancers compared to normal tissues (**Figure** **6**A; Figure S5A, Supporting Information), and these 37 Kla sites were an obvious enrichment of multiple molecular functions and protein complexes regulating RNA splicing, chromatin binding, protein binding, and transcription (Figure 6B‐D; Figure S5B, Supporting Information), indicating the potential functional significance of lysine lactylation in gene regulation. In addition, 46 common DEPs in GI tumors were also analyzed (Figure S5C, Supporting Information), and pathway analysis revealed that these proteins were enriched in many essential functional pathways (Figure S5D‐F, Supporting Information). For example, the ferroptosis pathway was enriched by both common differential Kla sites and DEPs in GI tumors (Figure S5G, Supporting Information).

As modification omics based on the common differential Kla sites in GI cancers emphasizes the dysregulation of gene expression, the 12 Kla sites in proteins associated with RNA splicing, chromatin binding, or transcription were further analyzed. Widespread Kla throughout the GI proteome indicated that these 12 Kla sites were among the top sites with high frequencies and fold changes between GI tumors and NATs (Figure 6E,F; Figure S5H, Supporting Information). These 12 Kla sites were also conserved among different species, some of which were located in the protein secondary structures (Figure 6G). In addition, seven of these 12 Kla sites were differentially expressed in tumors with diameter > 5 cm (Figure S5I, Supporting Information). Moreover, proteins with these 12 Kla sites were generally upregulated in four GI cancers (Figure S6A,B, Supporting Information). By correlating the lactylation levels of 12 sites with other profiling data, we found that tumors with higher lactylation showed proteomic downregulation of oxidative phosphorylation and upregulation of processes involving the spliceosome, DNA replication, DNA repair, and cell cycle. The lactylome showed that tumors with higher lactylation levels had Kla upregulation in the ribosome, spliceosome, glycolysis/gluconeogenesis, and pentose phosphate pathways (Figure S6C, Supporting Information). We further observed positive associations between BRDs, HAT1, and ELP3 protein levels and these 12 Kla sites, as well as negative associations between SIRT2 and SIRT3 protein levels and most differential Kla sites (Figure 6H,I). Collectively, these results show the consistency of Kla alterations among distinct GI cancers, suggesting that targeting these signatures may prove beneficial for future clinical treatments.

### 2.6 K10 Lactylation in CBX3 Promotes the Interaction of CBX3 with H3K9me3 and GI Tumor Growth

**Figure 7:**
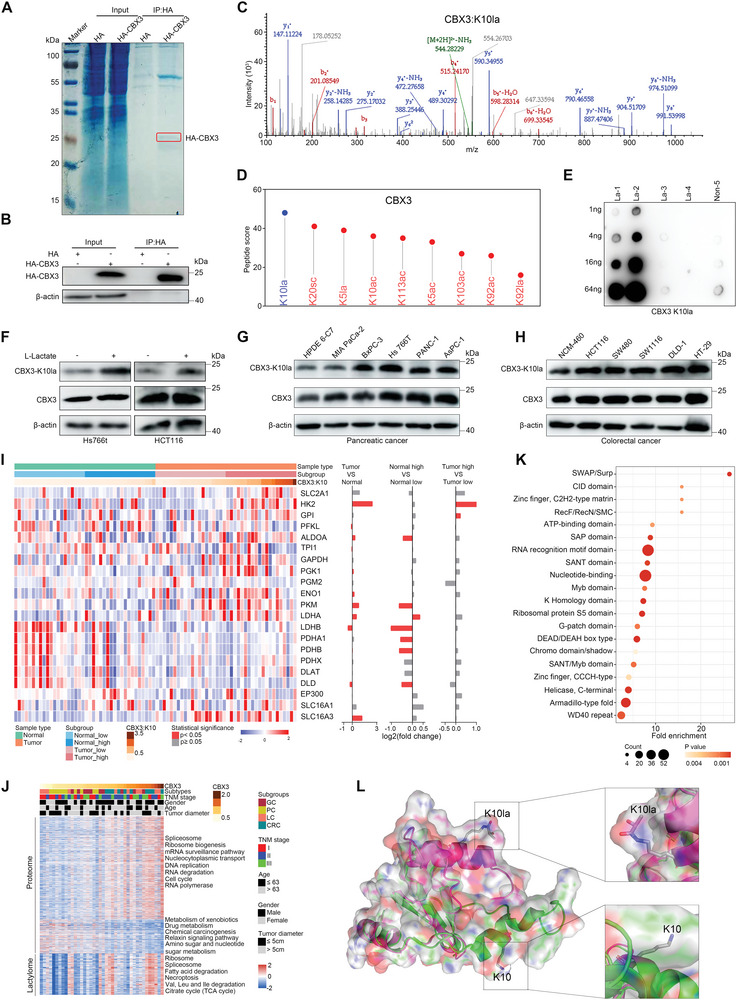
CBX3 K10 lactylation is commonly elevated in GI cancer cells. A–C) Proteins interaction with exogenous HA‐CBX3 in Hs766T cells was enriched by immunoprecipitation for LC/MS‐MS analysis. Representative spectra for peptide‐containing K10 lactylation is shown. The band y‐type product ions are indicated on each spectrum (C). D) Analysis of the abundance of lysine sites with different acylation modifications in CBX3. E) A rabbit polyclonal antibody was generated to specifically recognize the lactylated K10 in the peptide NKTTLQ‐K(Lactyl)‐MGKKQN. We first designed and synthesized different CBX3 peptides based on protein sequence for animal immunity, purification, and detection. The efficiency and specificity analysis of the anti‐CBX3‐K10la antibody using dot blots (La‐1, TTLQK(la)MGKKQNC; La‐2, NKTTLQK(la)MGKKQNC; La‐3, NKTTLQKMGK(la)KQNC; La‐4, NKTTLQKMGKK(la)QNC; and Non‐5, NKTTLQKMGKKQNC). F) Hs766T and HCT116 cells were pretreated with lactate (10 mM), and total CBX3 and the K10la levels were determined by Western blot. The quantitative analysis of CBX3‐K10la/CBX3 was shown in Figure S7H (Supporting Information) (n = 3 samples per group). G,H) Levels of total and lactylated CBX3 at lysine 10 in GI cancer and corresponding normal cells (HPDE6‐C7 and NCM‐460) were determined by Western blot. The quantitative analysis of CBX3‐K10la/CBX3 is shown in Figure S7I,J (Supporting Information) (n = 3 samples per group). I) Heatmap showing the proteomic expression of proteins involving the production and transport of lactate based on CBX3 K10la. The three columns of the histograms on the right show the differential expression analysis of each protein in indicated comparisons. *p* values were calculated using the two‐tailed Welch's t test. J) Associations of CBX3 density with clinicopathological characteristics and multiomics profiling. K) Protein domain analysis based on the proteins both correlated with CBX3 and its K10la level. L. Docking analysis of CBX3 structure with K10 lactylation or non‐lactylation, and K10 is shown with colored balls and sticks. See also Figure S7 (Supporting Information).

Recent studies have revealed that Kla modifications in histones are of great significance in gene expression.^[^ ^18^, ^22^ ^]^ Our lactylome data also emphasized the dysregulation of gene regulation in GI tumors, such as chromatin remodeling, which is tightly associated with cancer progression.^[^ ^23^, ^24^ ^]^ The common differential Kla sites involved in chromatin binding were further analyzed. The lactylation occurring for CBX3 K10 (CBX3 K10la), which is also located in one potential small‐molecule binding pocket of CBX3, was upregulated most obviously in GI cancers (Figure S7A‐D, Supporting Information). To confirm the occurrence of CBX3 K10la in GI cancer cells, the transiently expressed CBX3 was immunoprecipitated (**Figure** **7**A,B), and acylation modifications in CBX3 were analyzed by LC‐MS/MS (Figure 7C,D). In addition, a specific antibody recognizing CBX3 K10la was generated and verified using dot blot and ELISA (Figure 7E; Figure S7E,F, Supporting Information). Western blot analysis using this specific antibody showed that lactate promoted lactylation of CBX3 at K10, further confirming the existence of CBX3 K10la (Figure 7F; Figure S7G, H, Supporting Information). Moreover, CBX3 K10la levels were obviously increased in pancreatic and colorectal cancer cells compared with normal epithelial cells (Figure 7G,H; Figure S7I,J, Supporting Information). Consistently, IHC staining of human GI tumors and NATs with this antibody revealed a significantly higher signal intensity in GI cancer tissues (Figure S7K, Supporting Information). To clarify the factors that could affect cellular CBX3 K10la levels, the proteomic data of genes involved in the production and transport of lactate were investigated. GI tumor tissues with higher CBX3 K10la intensities exhibited elevated protein levels of hexokinase 2 (HK2), glucose‐6‐phosphate isomerase (GPI), and pyruvate kinase M (PKM) (Figure 7I). However, this was not observed in normal tissues, likely because cancer cells primarily depend on glycolysis for energy.^[^ ^25^ ^]^ In contrast, the protein levels of the two main subunits (PDHB and PDHX) of the pyruvate dehydrogenase complex were lower in NATs with higher CBX3 K10la intensity. In addition, the expression of solute carrier family 16 member 3 (SLC16A3), which mediates cellular lactate exchange, was much higher in tissues with higher CBX3 K10la intensity.

By examining the correlation of CBX3 and CBX3 K10la with other profiling data (Figure S6C, Supporting Information; Figure 7J), we found that tumors with higher CBX3 K10la showed proteomic downregulation of metabolism of oxidative phosphorylation, and upregulation of processes involving the spliceosome, DNA replication, and the cell cycle. Lactylome analysis showed that tumors with higher CBX3 K10la levels had Kla upregulation in ribosome, spliceosome, and metabolic pathways. In addition, proteins significantly related to both CBX3 and its K10la were enriched in multiple protein domains responsible for gene regulation (Figure 7K). In addition, the molecular docking analysis of CBX3 with K10la showed that K10 lactylation affected the conformation of CBX3 (Figure 7L). These results suggest a potential role for lactylation at K10 in mediating the function of CBX3 and regulating gene expression during GI cancer progression.

**Figure 8:**
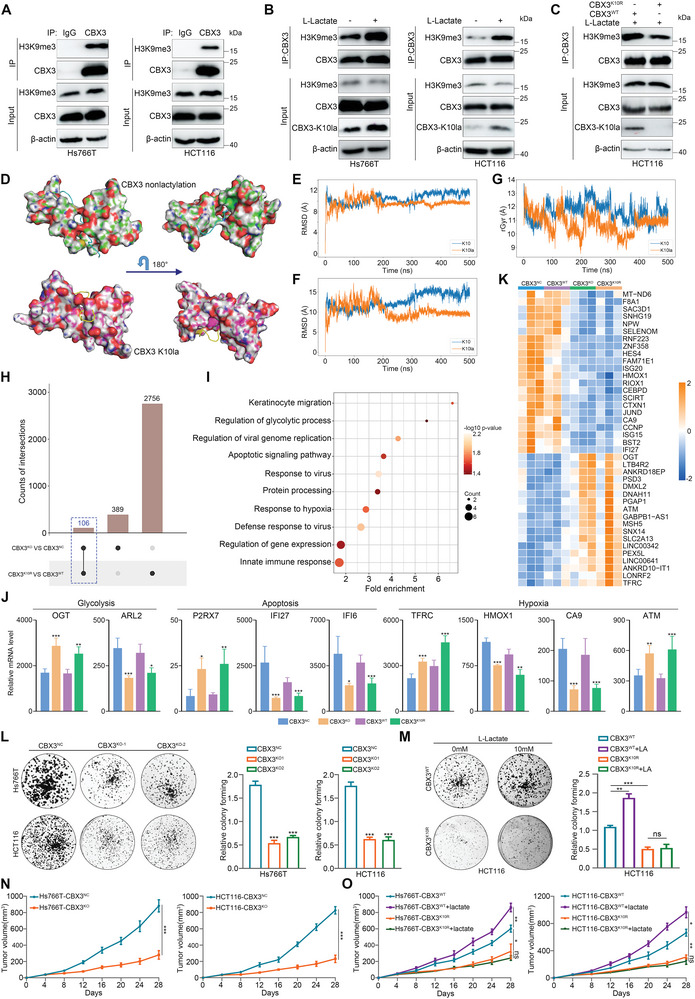
K10 lactylation promotes the interaction of CBX3 with H3K9me3 and GI tumor growth. A) Co‐immunoprecipitation assay using CBX3 as bait protein with IgG as negative control showed the interaction between CBX3 and H3K9me3 (n = 3 biological replicates). B) GI cancer cells were treated with lactate, lysed with weak RIPA lysis, and co‐immunoprecipitated with CBX3 antibody (n = 3 biological replicates). C) Co‐immunoprecipitation assays were performed using CBX3^WT^ and CBX3^K10R^ cells treated with lactate. The quantitative analysis of relative enrichment of H3K9me3 is shown in Figure S8F (Supporting Information) (n = 3 samples per group). D) Docking analysis of nonlactylated or K10‐lactylated CBX3 with H3K9me3 peptide based on a CBX3‐H3K9me3 complex conformation in the PDB database (PDB: 2L11). E) Backbone root‐mean‐square deviation (RMSD) of nonlactylated and K10‐lactylated CBX3 as a function of time. ns, nanosecond. F) RMSD of H3K9me3 peptide in nonlactylated or K10‐lactylated CBX3 as a function of time. G) Radius of Gyration (rGyr) analysis of H3K9me3 peptide in nonlactylated or K10‐lactylated CBX3. H) Intersection of DEGs identified from CBX3^KO^ or CBX3^K10R^ cells by RNA‐seq analysis (n = 3 samples per group). I) Pathway enrichment analysis based on the DEGs identified from both in CBX3^KO^ and CBX3^K10R^ cells. J) Differentially expressed genes both in CBX3^KO^ and CBX3^K10R^ cells, and their associated biological pathways. K) Hierarchical clustering of DEPs in CBX3^KO^ and CBX3^K10R^ cells (adjusted *p* < 0.01). L) Colony formation analysis of the effect of CBX3 knockout on Hs766T and HCT116 cell proliferation (n = 3 samples per group). M) Cell proliferation by colony formation assay in CBX3^WT^ and CBX3^K10R^ cells with treatment of lactate (n = 3 samples per group). N) Nude mice were hypodermically injected with CBX3 silent GI cancer cells. Tumor volumes were measured during the growth process (n = 6 samples per group). O) Nude mice were hypodermically injected with CBX3^WT^ and CBX3^K10R^ cells treated with lactate. Tumor volumes were measured during the growth process (n = 4 samples per group). Data are presented as mean ± SEM (J, L‐O). *p* values were calculated by two‐sided Wilcoxon rank‐sum test (J), one‐way ANOVA with Dunnett's multiple comparison test (L, M), two‐way ANOVA with Bonferroni's multiple comparison test (N), and two‐way ANOVA with Dunnett's multiple comparison test (O). *p < 0.05, **p < 0.01, and ***p < 0.001. ns indicates non‐significant. See also Figures S8 and S9 (Supporting Information).

CBX3 is a highly conserved heterochromatin protein that specifically interacts with chromatin by binding to H3K9me3 and regulates gene silencing.^[^ ^26^ ^]^ This result was further supported by co‐immunoprecipitation analysis in our study, which showed that endogenous CBX3 bound to H3K9me3 in GI cancer cells (**Figure** **8**A; Figure S8A, Supporting Information). As K10 is located near the chromodomain (CD) of the CBX3 protein^[^ ^27^ ^]^ (Figure S8B, Supporting Information), and considering that CD is responsible for binding H3K9me, we immunoprecipitated CBX3 in GI cancer cells treated with lactate and demonstrated that lactate could promote the interaction of CBX3 with H3K9me3 (Figure 8B). In addition, we employed CRISPR/Cas9 technology to knock out endogenous CBX3 in GI cancer cells (Figure S8C, Supporting Information), and the CBX3‐depleted cells were then transfected with lentiviral expression plasmids encoding WT CBX3 (CBX3^WT^) or its mutant K10R (CBX3^K10R^), in which Lys10 was replaced with arginine (Figure S8D, Supporting Information). Co‐immunoprecipitation confirmed the binding of H3K9me3 to WT CBX3, and to a much lesser extent to CBX3^K10R^ (Figure 8C; Figure S8E,F, Supporting Information), indicating that lactylation at K10 promoted the binding of CBX3 to H3K9me3.

To better understand how lactylation promotes the interaction between CBX3 and H3K9me3, we used molecular dynamic simulations to compare the structural dynamics of nonlactylated and K10‐lactylated CBX3, and analyzed the binding status of the H3K9me3 peptide (Figure 8D; Figure S8G, Supporting Information). Docking analysis showed that nonlactylated CBX3 conferred a more dynamic conformation (with convergence starting at 300 ns), whereas K10 lactylation remained stable starting at 200 ns (Figure 8E). Moreover, the root‐mean‐square deviation (RMSD) of the H3K9me3 peptide in lactylated CBX3 fluctuated at 7.5 Å‐10 Å, which was much lower than in nonlactylated CBX3 (12.5 Å‐15 Å) (Figure 8F). In addition, the rotary radius of the H3K9me3 peptide in lactylated CBX3 was much smaller than that in nonlactylated CBX3 (Figure 8G), indicating that the fluctuation amplitude decreased during simulation process of the lactylated CBX3 structure. These results suggest that CBX3 lactylation at K10 induces conformational changes favorable for binding to the H3K9me3 peptide.

To explore the correlation between CBX3 K10la and gene expression, we performed RNA‐seq in GI cancer cells with CBX3 deleting or CBX3^K10R^ transfection (Figure S8H, Supporting Information). Bioinformatics analysis revealed 3251 and 615 differentially expressed genes (DEGs) in the CBX3^KO^ and CBX3^K10R^ cell lines, respectively (Figure S8I,J, Supporting Information). Intersection analysis of the DEGs between the two above groups demonstrated that 106 genes were dysregulated in both CBX3^KO^ and CBX3^K10R^ cells (Figure 8H). KEGG pathway analysis indicated that common DEGs were distributed in many hallmark cancer pathways, including glycolysis, apoptosis, and response to hypoxia (Figure 8I,J). In addition, more stringent unsupervised hierarchical clustering of common DEGs (*p* < 0.01) almost entirely distinguished WT CBX3 from CBX3^KO^ and CBX3^K10R^ (Figure 8K). These results suggested that Kla can regulate the transcription process mediated by CBX3, which is critical for GI cancer progression. Consistent with TCGA pan‐cancer data (Figure S6A, Supporting Information), our proteomics data indicated that the abundance of CBX3 was globally elevated in GI cancers (Figure S9A, Supporting Information). Kaplan‐Meier survival analysis revealed that the expression of CBX3 was negatively correlated with the prognosis of patients with GI cancer (Figure S9B, Supporting Information). Here, we investigated the function of CBX3 and its lactylation in GI cancer progression. Deletion of CBX3 induced significant proliferation arrest in GI tumors (Figure 8L; Figure S9C, Supporting Information), and CBX3^K10R^ restoration exhibited significantly slower growth than CBX3^WT^ (Figure 8M; Figure S9D‐F, Supporting Information). In addition, GI cancer cells with CBX3 deletion or CBX3^K10R^ transfection were injected into nude mice subcutaneously. CBX3 depletion or CBX3^K10R^ expression impeded tumor growth (Figure 8N,O; Figure S9G‐J, Supporting Information). These results indicated that the lactylation on CBX3 was critical for GI cancer progression.

## 3 Discussion

The discovery and functional identification of molecular signatures in GI cancers, accelerated by high‐throughput proteogenomic analyses, have advanced our mechanistic understanding of tumor progression.^[^ ^28^, ^29^, ^30^, ^31^ ^]^ Currently, most targeted therapies are restricted to specific tumor types, and there is an urgent need to expand the exploration of the mechanistic correlates of oncogenic driver alterations across distinct GI tumor types.

Enhanced glycolysis and lactate accumulation are common features of various cancers,^[^ ^25^, ^32^, ^33^ ^]^ and intracellular lactate drives a recently described type of protein modification, lysine lactylation.^[^ ^18^ ^]^ A previous lactylome analysis of hepatitis B virus‐related hepatocellular carcinoma (HCC) has broadened our knowledge of the molecular events associated with this fatal malignancy.^[^ ^34^ ^]^ However, whether and how protein functions are regulated by lactylation modifications require further elucidation, especially regarding the molecular dynamics of Kla regulation in distinct GI tumors. Here, we adapted a high‐throughput proteomics workflow to systematically identify Kla across distinct GI samples, which is the first characterization and quantitative profiling of Kla in multiple GI tumors. A large set of Kla sites was identified in GI tumors, and most were found on non‐histones involved in numerous hallmark processes. Although GI cancer is a very heterogeneous disease at the cellular and histological levels, comprehensive lactylome analysis of paired tumors and NATs revealed the activation status of functional pathways and clinically relevant signatures. The changes in metabolic pathways mediated by dysregulated Kla modifications in GI tumors were consistent with a previous conclusion in HCC.^[^ ^34^ ^]^ In addition to metabolic alterations, multiple Kla sites in proteins involved in RNA regulation, genome stability, and proteasome are also dysregulated in GI cancer. This observation led us to speculate that these lactylproteins undergo biochemical and structural alterations based on Kla modifications, which significantly influence their functions.

In addition, clustering analysis of distinct GI cancers based on changes in Kla sites or proteins provides insights into the molecular dynamics of oncogenic changes, revealing potential therapeutic avenues for single to four GI cancers. Moreover, intersection analysis of the differential Kla sites in the four types of GI cancer revealed several sites that were dysregulated in specific GI cancers and enriched in many functional processes, which may provide unique therapeutic opportunities. Importantly, 37 Kla sites were differentially expressed in all four GI tumors, and most of these lactylproteins were involved in gene regulation, including spliceosome, chromatin remodeling, and transcription. In humans, nearly 95% of genes undergo alternative splicing to produce transcripts and increase their proteome complexity.^[^ ^35^, ^36^, ^37^ ^]^ The splicing process is catalyzed by the spliceosome, a ribonucleoprotein complex responsible for binding pre‐mRNA^[^ ^38^ ^]^ that governs multiple aspects of cellular activities and has been implicated in several human cancers.^[^ ^39^, ^40^, ^41^, ^42^ ^]^ Widespread protein modifications have been confirmed to occur in spliceosome proteins,^[^ ^43^, ^44^, ^45^ ^]^ however, the functions and mechanisms of RNaA splicing regulated by lactylation modifications in GI cancer have rarely been reported. Our lactylome analysis revealed common differential Kla sites specifically enriched in RNA splicing. These sites were also conserved among different species, indicating a potentially critical role for RNA splicing mediated by lactylation modification in GI cancer. In addition, some transcription factors, including HDGF, NCL, PCBP1, and PCBP2, were also found to be largely modified by lysine lactylation in all four types of GI tumors, and further confirming the regulation of Kla in gene expression.

Chromatin remodeling is one of the main epigenetic mechanisms of gene expression regulation.^[^ ^46^, ^47^ ^]^ CBX3, a highly conserved heterochromatin protein that specifically recognizes H3K9me3, is involved in the regulation of heterochromatin formation.^[^ ^48^ ^]^ However, the influence of CBX3 lactylation on gene expression has not yet received attention. Comparative lactylome analysis revealed that the level of CBX3 K10la was much higher in all GI cancers, which was verified using a specific antibody. We also found that GI tumors with higher CBX3 lactylation showed proteomic downregulation of oxidative phosphorylation metabolism. Previous studies have demonstrated that glucose uptake and lactate production are higher in cancer cells than in normal tissues, and a smaller fraction of this glucose is utilized by oxidative phosphorylation,^[^ ^49^ ^]^ which provides cancer cells with adequate energy. Lactate and acetyl‐CoA are mainly generated from pyruvate and played opposite roles in the glycolytic switch.^[^ ^50^ ^]^ Notably, both lactylation and acetylation prefer lysine (Lys) as the residue mediating multiple biological processes, which could be considered a reflection of the metabolic state and tumor cells fate. The elevated glycolysis leads to lactate accumulation and CBX3 K10 lactylation in GI cancers, and oxidative phosphorylation may be inhibited owing to decreased acetyl‐CoA production. Moreover, we demonstrated for the first time that K10la is essential for maintaining CBX3 interaction with H3K9me3 and further regulates cancer hallmark process‐gene expression and GI progression. In summary, this study highlights a number of Kla modifications that are worthy of further investigation to achieve a comprehensive understanding of protein regulation across distinct GI cancers.

## 4 Experimental Section

### Human Samples of GI Cancer

Histologically confirmed GI cancer and adjacent normal tissue samples for lactylome and proteome analyses were obtained from Qilu Hospital (KYLL‐202301‐017), Shandong Provincial Qianfoshan Hospital (YXLL‐KY‐2022(068)), and The Second Hospital of Shandong University (KYLL‐2021(KJ)P‐0021), Jinan, China. The protocol for proteomic analysis of the samples was approved by the institutional research ethics review board. The clinicopathological characteristics of the patients and their tumors are summarized in Table S1 (Supporting Information). The prospective cohort consisted of 65% male and 35% female patients with a median age of 63 years. Histologically, 30% of patients were in early stage I, 40% in stage II, and 30% in stage III (Figure S1A, Supporting Information). Surgically resected tissue samples were frozen in liquid nitrogen and stored at −80 °C before use. GI tumor and adjacent normal tissue samples for IHC analysis were collected from Shandong Cancer Hospital and Institute, Jinan, China, with approval from the Research Ethics Committee of the hospital and consent from all participants.

### Sample Preparation for LC‐MS/MS

All tissues were washed with 1× phosphate‐buffered saline (PBS) buffer to exclude the disturbance of high‐abundance protein in the blood, and ground with liquid nitrogen into cell powder. Then, the samples were added with four volumes of lysis buffer supplemented with 1% Triton X‐100, 1% protease inhibitor cocktail, 3 µM trichostatin A, and 50 mM nicotinamide, followed by sonication three times on ice using a high intensity ultrasonic processor (Scientz), and the protein was collected by centrifugation at 12 000 g at 4 °C for 10 min. The supernatant was collected and the protein concentrations were determined using a Bio‐Rad BCA protein assay kit.

### Trypsin Digestion and Pan Antibody‐Based Lactylation Enrichment

For digestion, the protein samples were reduced using 5 mM dithiothreitol for 30 min at 56 °C, then alkylated using 11 mM iodoacetamide for 15 min at room temperature in the dark, then diluted by adding 100 mM TEAB to urea concentration of <2 M. Proteins were digested at 1:50 trypsin‐to‐protein mass ratio for the first digestion at 37 °C overnight and 1:100 trypsin‐to‐protein mass ratio for a second 4 h‐digestion. Finally, the eluted peptides were purified using a C18 solid‐phase extraction column and freeze‐dried using a SpeedVac device for further use.

To enrich Kla‐modified peptides, tryptic peptides dissolved in NETN buffer (100 mM NaCl, 1 mM EDTA, 50 mM Tris‐HCl, and 0.5% NP‐40; pH 8.0) were incubated with prewashed antibody beads at 4 °C overnight with gentle shaking. The beads were then washed four times with NETN buffer and twice with H_2_O. Bound peptides were eluted from the beads using 0.1% trifluoroacetic acid. Finally, the eluted fractions were combined and vacuum‐dried. For LC‐MS/MS analysis, the resulting peptides were desalted using C18 ZipTips (Millipore), according to the manufacturer's instructions.

### LC‐MS/MS‐Based Analysis of Peptides

The peptides were dissolved in solvent A (0.1% formic acid, 2% acetonitrile in water), and directly loaded onto a homemade reversed‐phase analytical column (25 cm length, 100 µm i.d.) for both lactylome and proteomic analyses. The enriched Kla‐peptides were separated with a gradient from 7% to 24% solvent B (0.1% formic acid in acetonitrile) for 42 min, 24% to 32% in 12 min, increasing to 80% in 3 min, and then holding at 80% for the last 3 min, all at a constant flow rate of 450 nL min^−1^ on a nanoElute ultra‐high‐resolution liquid chromatography (UHPLC) system (Bruker Daltonics). For proteomic analysis, the tryptic peptides were separated with a gradient from 6% to 24% solvent B (0.1% formic acid in acetonitrile) over 70 min, 24% to 35% over 14 min, increasing to 80% over 3 min, and then held at 80% for the last 3 min, all at a constant flow rate of 450 nL min‐1 on a nanoElute UHPLC system. All eluted peptides were subjected to a capillary source, followed by timsTOF Pro (Bruker Daltonics) MS. Precursors and fragments were analyzed using a TOF detector with an MS‐MS scan range from 100 to 1700 m/z. The timsTOF Pro was operated in the parallel accumulation serial fragmentation (PASEF) mode. Precursors with charge states of 0–5 were selected for fragmentation, and ten PASEF MS‐MS scans were acquired per cycle. The dynamic exclusion was set at 30 s.

### Database Search

The acquired MS and MS/MS data were searched against the human SwissProt database (20 422 entries) concatenated with a reverse decoy database using the MaxQuant search engine (version 1.6.15.0).^[^ ^51^ ^]^ Search parameters were set, including the proteolytic enzyme trypsin, and allowed for up to two missed cleavage sites. The precursor ion tolerance was set to 20 ppm, main search peptide tolerance to 5 ppm, and mass tolerance of fragment ions to 0.02 Da. Carbamidomethylation on Cys was specified as a fixed modification, and acetylation at the protein N‐terminus and oxidation on Met were specified as variable modifications. For the analysis of the Kla‐modified peptides enriched data, the set of variable modifications included lysine lactylation. The false discovery rates (FDRs) were set at <1% for peptide‐spectrum matches and sites and protein identifications.

The intensity‐based quantification of protein groups was extracted from the MaxQuant result files to represent the expression of a particular protein across samples. The MaxLFQ algorithm in the MaxQuant software was used to quantify the protein levels across all samples. The Kla‐peptide levels were calculated based on the raw spectral intensity. A catalog of all confidently identified peptides with lactylation modifications was constructed and normalized based on their protein abundance to exclude the level changes of Kla‐peptides caused by the dynamics of protein levels and compared between tumor and adjacent tissues.

### Basic Characteristic Analysis of Kla in GI Samples

A total of 11698 Kla sites on 3156 proteins were identified in the GI tumors and NATs, among which 6260 Kla sites were identified in the liver, 3763 in the pancreatic, 8100 in the colorectal, and 5526 in the gastric tissues. The Kla sequence motif was generated using the Motif‐X algorithm with the human proteome as the background. Subcellular localization of lactylproteins was analyzed using WoLFPSORT program.^[^ ^52^ ^]^ Protein classification was performed based on the detailed Gene Ontology analysis using the DAVID database.^[^ ^53^, ^54^ ^]^

### Differential Markers Analysis

Protein and lactylation abundance were compared between GI tumors and adjacent samples using the Wilcoxon rank‐sum test. Significant signatures were made based on an adjusted *p* < 0.05, with a >1.5‐fold abundance change in each comparison, indicating upregulated and downregulated proteins or lactylsites. We also developed linear models to identify differential markers between two key variables, including tumor stage and tumor diameter. A total of 103 differential Kla sites and 762 DEPs were identified that could distinguish histopathologically aggressive from stage I cancers, whereas 401 differential Kla sites and 252 DEPs were identified that were associated with tumor diameter (adjusted *p* < 0.05).

In addition, differentially expressed proteins and Kla sites identified in these comparisons were subjected to pathway overrepresentation analysis. It is particularly important to note that there were multiple sites on the same protein whose *p* values were all <0.05, we chose the site with the largest log2 ratio (between groups in all comparisons) for analysis.

### Functional Enrichment Analysis of Proteomics Data

To gain further insight into the biological implications, pathway enrichment analysis based on lactylation or protein abundance was performed using single sample gene set enrichment analysis (ssGSEA)^[^ ^55^ ^]^ based on the MSigDB database. The ssGSEA implementation is available at https://github.com/broadinstitute/ssGSEA2.0.

### Cell Lines and Cell Culture

HEK293T, Hs766T, BxPC‐3, PANC‐1, AsPc‐1, MIA PaCa‐2, NCM460, HCT116, SW1116, SW480, HT‐29, and DLD‐1 cells were obtained from the American Type Culture Collection (ATCC). The cells were grown in high glucose Dulbecco's modified Eagle medium (DMEM; Biological Industries) supplemented with 10% fetal bovine serum (FBS; Biological Industries). All cultures were maintained at 37 °C under 5% CO_2_.

### Expression Plasmids and Mutagenesis

The PCR‐amplified human *CBX3* gene fragment was cloned into the pLVX‐AcGFP‐N1 and pcDNA6B‐HA vectors. The CBX3‐K10R mutant was generated using a QuikChange site‐directed mutagenesis kit (FM111‐01; TransGen Biotech). All plasmid cloning experiments were performed using Stbl3 (BC108‐01; Biomed) or DH5α (BC102‐02; Biomed) strains. Lipofectamine 3000 (Invitrogen) was used to transfect the vectors. Primers used were as follows: CBX3‐F, 5′‐ATGGCCTCCAACAAAACTACATTG‐3′; and CBX3‐R, 5′‐TTGAGCTTCATCTTCTGGACAAGAA‐3′; K10R‐F, 5′‐ AAAACTACATTGCAACGAATGGGAAAAA‐3′; and K10R‐R, 5′‐ TCGTTGCAATGTAGTTTTGTTGGAGGCC‐3′.

### CBX3 KO by CRISPR/Cas9 System

CBX3‐KO cell lines were generated using CRISPR/Cas9‐mediated gene targeting. Single‐guide RNAs (sgRNAs) targeting exon 3 of *CBX3* (5′‐CACCGGAGAGCCTGAAGAATTTGTCG‐3′, 5′‐CACCGGTTTTCCACGACAAATTCTTC‐3′, 5′‐CACCGGAAAGAGTAAAAAAGTTGAAG‐3′) were designed to target the *CBX3* gene and ligated into the PX330 vector, following transient transfection the pX330‐U6‐gRNA‐Cas9 expression vector with Lipofectamine 3000. G418 (ST081; Beyotime) was added after 48 h, and a single cell expressing green fluorescent protein (GFP) was selected with flow cytometry sorters and cultured in a 96‐well plate. The targeted cleavage was Sanger‐sequenced in pMD‐19T vector with the PCR product of CBX3‐deficient colonies (forward primer: 5′‐CCCTGGGATATAGAAGTAAA‐3′; reverse primer: 5′‐CTGTAGGTGTCTTTAAATG‐3′).

### Establishment of Stable Cell Lines with Lentivirus

The constructed plasmids were transfected into HEK293T cells, together with the package plasmids psPAX and PMD2.G, at a ratio of 4:3:1, using the Lipofectamine 3000 transfection reagent. The supernatants were collected and filtered through a 0.22 µM filter membrane. The cells were transfected with the collected lentivirus particles and selected by puromycin (P8230; Solarbio) for two weeks. The establishment of stable cell lines was verified by Western blot.

### Mouse Experiments

All animal experiments were approved by the Shandong University Animal Care and Use Committee (ECSBMSSDU2023‐2‐36) and performed in accordance with the institutional guidelines. The procedures for monitoring tumor growth have been described previously.^[^ ^56^ ^]^ Suspensions containing 5 × 10^6^ cells were injected subcutaneously into the flanks of 4‐week‐old nude mice. Tumor volumes were measured every 4 days and calculated as follows: (large diameter × smaller diameter^2^)/2. All the mice were housed in a specific pathogen‐free laboratory animal room.

### Immunoblotting and Immunoprecipitation

The cells exposed to different conditions were lysed in RIPA lysis buffer (Beyotime) containing a protease inhibitor cocktail (0 469 313 2001; Sigma‐Aldrich). The protein concentration was determined using a BCA Protein Assay Kit (A53225; Thermo Scientific). For immunoprecipitation experiments, the whole‐cell lysates were immunoprecipitated with anti‐HA agarose (A2095; Sigma‐Aldrich) overnight at 4 °C. Beads were washed 3 times with wash buffer and samples were separated by SDS‐PAGE. Western blot was performed as described previously^[^ ^57^ ^]^ with the following antibodies: HA (1:2000, H3663, Sigma‐Aldrich), CBX3 (1:1000, sc‐398562, Santa Cruz), H3K9me3 (1:1000, PTM‐616, PTM BIO), or β‐Actin (1:1000, AB0035, Abways) at 4 °C overnight. The anti‐lactyl‐lysine 10 specific polyclonal antibody against CBX3 (CBX3‐K10la) was made by Jingjie PTM BioLab Co., Inc.

### Cell Proliferation Assays

The cells were incubated in 6‐well plates and maintained in DMEM. The medium was replaced every three days. After approximately 2 weeks, the cells were collected after being washed twice with PBS and fixed in 4% paraformaldehyde for 30 min. Finally, the cells were stained with 0.1% crystal violet. The visible colonies were photographed and counted.

### IHC

IHC staining was performed on the tissues according to previously published protocols.^[^ ^57^ ^]^ Dissected tumors were fixed in 4% paraformaldehyde overnight and subsequently embedded in paraffin wax. Next, 4 µm thick sections were stained with indicated antibodies. Immunohistochemical analysis was performed for different markers in these arrays as described previously.^[^ ^57^ ^]^

### Molecular Dynamic Simulation

Docking between human K10‐lactylated CBX3 and the H3K9me3 peptide was modeled based on the CBX3‐H3K9me3 complex conformation (PDB: 2L11) using a Homology Model in Molecular Operating Environment (MOE). The molecular dynamics simulations were performed and adjusted using the Desmond (Maestro‐Desmond Interoperability Tools, Schrödinger) program based on the OPLS4 force filed.^[^ ^58^ ^]^ The protonation states of the residues in CBX3 and the ligands were assigned at pH 7.0. The complexes were solvated in a cubic box of TIP3P water models and NaCl was added to neutralize the system. The system was subjected to 100 ps minimization (Brownian motion simulation) to adequately equilibrated the complexes and solvent molecules. The solvated complex was equilibrated by carrying out a short minimization, 100 ps of heating with a Nose–Hoover chain thermostat, and 100 ps of density equilibration with an isotropic Martyna–Tobias–Klein barostat, followed by 500 ps of constant‐pressure equilibration at 300 K and 1 atm. The simulation results, including the root‐mean‐square deviation and rotary radius, were calculated with a cutoff of 9.0 Å and the time step was set to 2.0 fs. The trajectories were saved every 20 ps for further analysis.

### RNA‐Sequencing

Total RNA was extracted from the indicated cells using the TRIzol reagent (Magen) according to the manufacturer's instructions. The RNA concentration was determined based on the A260/A280 absorbance ratio, and the RNA RIN was determined using an Agilent Bioanalyzer 4150 system (Agilent Technologies, CA, USA). Only the qualified samples were used for library construction. Paired‐end libraries were generated using the ABclonal mRNA‐seq Lib Prep Kit (ABclonal, China), and the mRNA was purified from 1 µg total RNA using oligo (dT) magnetic beads followed by fragmentation carried out using fragmentation buffer. Subsequently, first‐strand cDNAs were synthesized with random hexamer primers, followed by second‐strand cDNA synthesis using DNA polymerase I, RNAseH, buffer, and dNTPs. The synthesized double‐stranded cDNA fragments were then adapter ligated to prepare a paired‐end library. The adaptor‐ligated cDNA was used for PCR amplification. The PCR products were purified (AMPure XP system), and library quality was assessed using an Agilent Bioanalyzer 4150 system. Library preparations were sequenced on an Illumina Novaseq platform to generate 150 bp paired‐end reads. Quality control of the raw data in the fastq format was first performed using in‐house Perl scripts. In this step, remove the adapter sequence and filter out low quality (low quality, the number of lines with a string quality value less than or equal to 25 accounts for more than 60% of the entire reading) and N (N means that the base information cannot be determined) ratio that is greater than 5% reads to obtain clean reads. All subsequent analyses were based on clean data with high quality.

### Statistical Analysis

All statistical analyses were performed using GraphPad Prism 8.0 and R Statistical Computing Software. The relationship between a categorical variable and a quantitative variable was estimated with the Wilcoxon rank‐sum (two categories). The relationship between two quantitative variables was estimated with Spearman's correlation coefficients as indicated. *p* values were corrected for multiple hypothesis testing as specified in the text. Two‐sided log‐rank test analyses were used for the comparison of Kaplan‐Meier survival curves. The repeatability of the findings was confirmed by performing all experiments at least three times. Data are presented as the mean ± standard error of the mean (SEM). All data were evaluated for significance with appropriate multiple comparison tests. Adjusted *p* value < 0.05 was considered statistically significant. Significance levels are as follows: *p < 0.05, **p < 0.01, ***p < 0.001, and ****p < 0.0001; ns indicates non‐significant.

## Conflict of Interest

The authors declare no conflict of interest.

## Author Contributions

Y.D. and H.Z. contributed equally to this work. Y.W., G.W., and Y.D. devised and coordinated the project. Y.D. performed the analyses and quality control of the MS data, performed most of the experiments, and prepared the figures with the help of H.Z., Q.W., B.L., H.G., and D.L. Q.X., X.G., and Z.L. provided clinical specimens and patient information. P.G. provided pathological expertise. G.W. directed the proteomic experiments, guided the bioinformatics analysis, and wrote the manuscript. Y.W. conceived, designed, and oversaw the study, evaluated the data, and wrote the manuscript.

## Supporting information

Supporting Information

Supplemental Table 1

Supplemental Table 2

Supplemental Table 3

Supplemental Table 4

Supplemental Table 5

Supplemental Table 6

## Data Availability

The data that support the findings of this study are available from the corresponding author upon reasonable request.
