# Supplementary material for: Integrated Lactylome Characterization Reveals the Molecular Dynamics of Protein Regulation in Gastrointestinal Cancers
Source: Adv Sci (Weinh). 2024 Jul 17;11(35):2400227. doi: 10.1002/advs.202400227 (PMC11425215; doi:10.1002/advs.202400227)
Supplement: Supplementary file 1 — Supporting Information [file ADVS-11-2400227-s001.docx]

**SUPPLEMENTAL INFORMATION**

Supplemental Figures 1 to 9

**Figure S1**


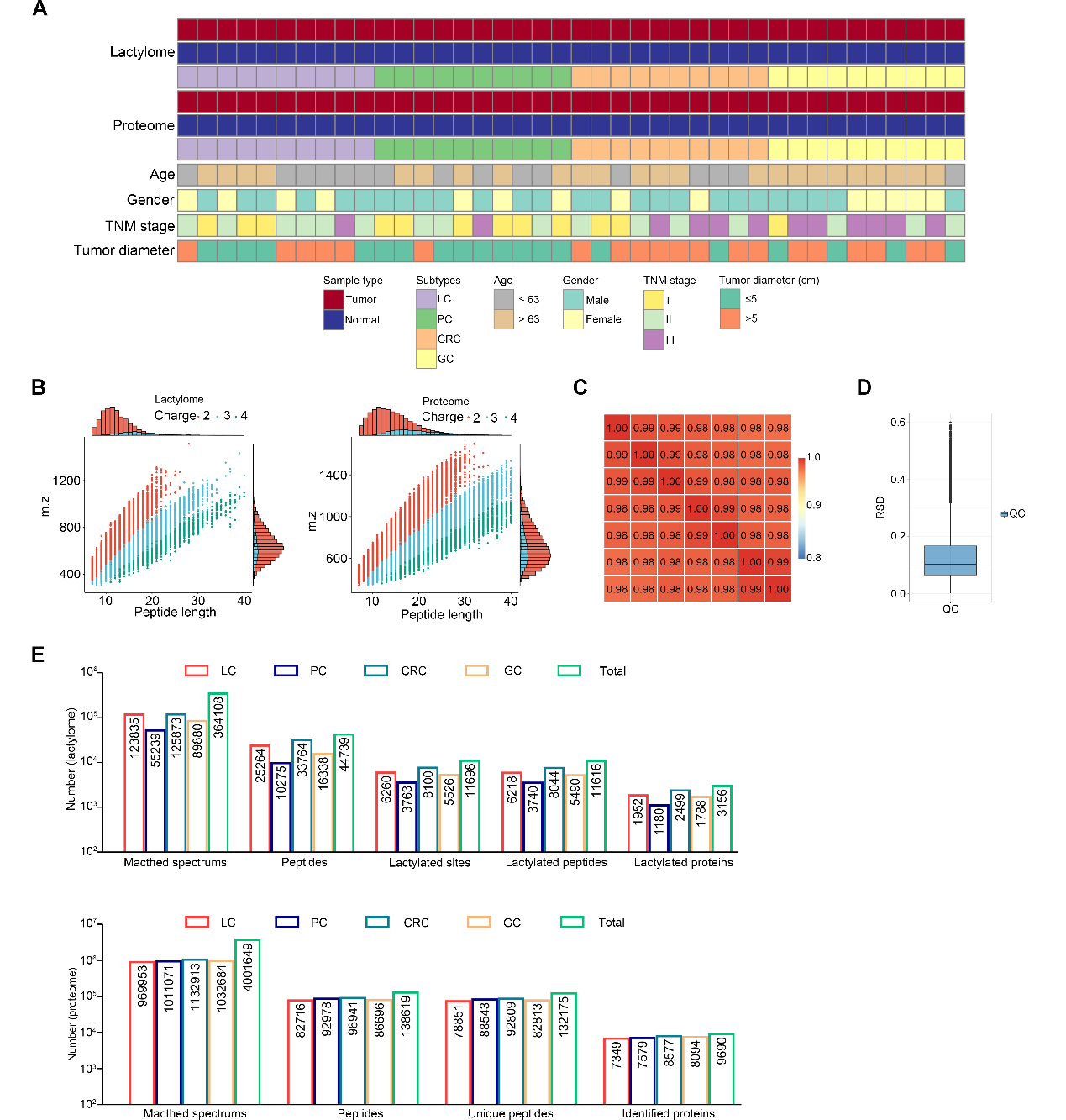


**Figure S1,** related to **Figure 1**. **Overview of the research pipeline and quality control for the mass spectrometry platform.**

**A.** Illustration of 40 cases of patients with GI cancer (liver, pancreatic, colorectal or gastric cancer) (n=10 for each type of cancer) included in the individual omics profiling. The proteome and lactylome experiments are shown, respectively. The main clinical information of each patient is shown. **B.** Distribution of mass-to-charge ratio (m.z) and length of differently charged peptides. **C, D.** Spearman correlation coefficients (**C**) and RSD (**D**) calculated from the lactylome analysis of each biological replicate used for quality control. **E.** Numbers of the peptides, Kla sites, lactylated peptides, and proteins identified from each type of GI sample.

**Figure S2**

**
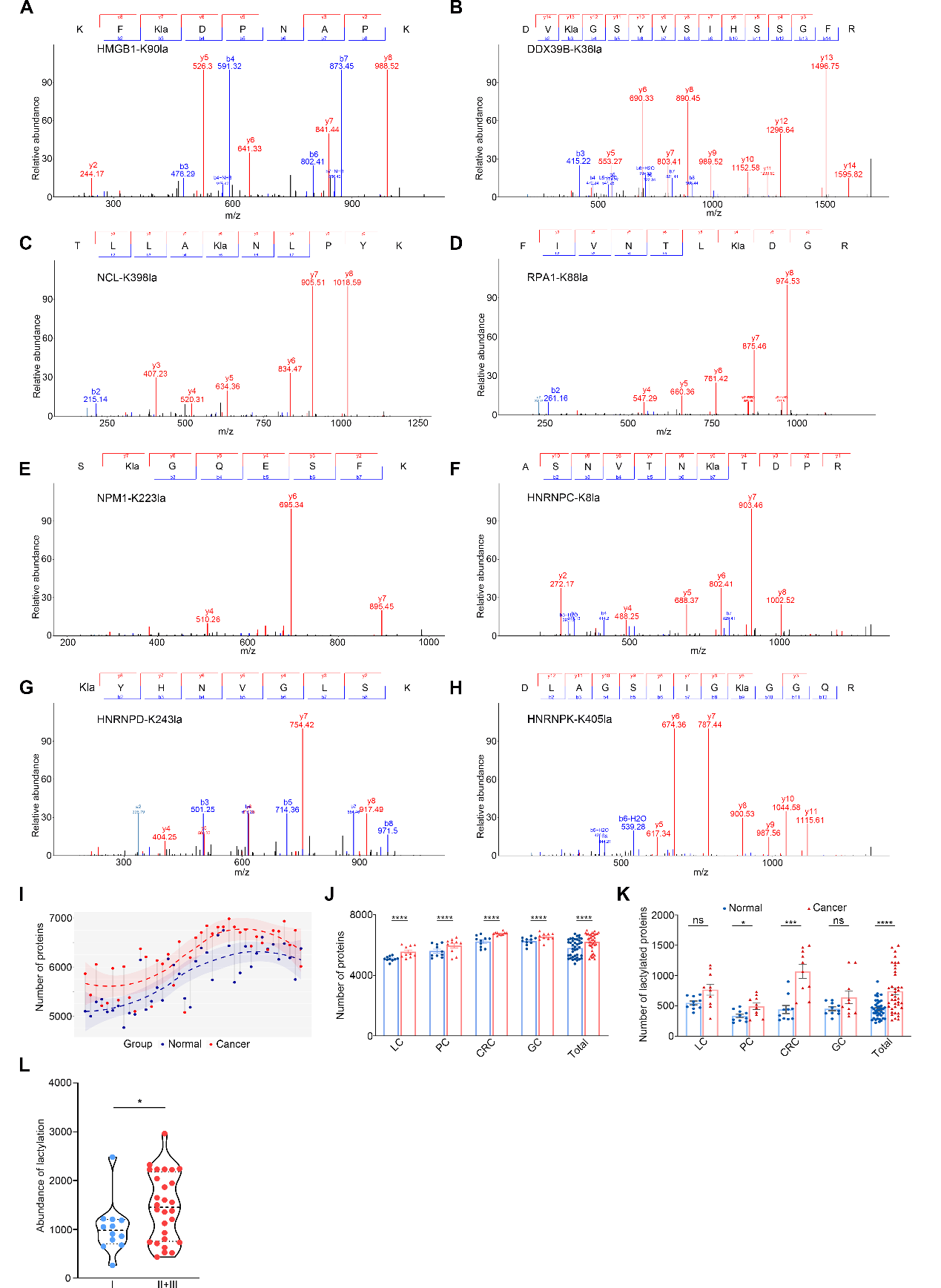
**

**Figure S2,** related to **Figure 1**. **Widespread Kla modifications identified in GI cancer.**

**A-H.** Representative spectra for different peptides-containing lactylation are shown. The band y-type product ions are marked on each spectrum. **I.** Overview of the protein identifications in GI tumor (red, n=40) and non-tumor (blue, n=40) samples. Pairwise samples are annotated with grey straight lines. The dashed curves were fitted using lasso regression and the shading that underlies the lasso curves denotes the 95% confidence intervals. **J, K.** Box plots of the protein and lactylated proteins identifications in the GI samples. (LC, n=10; PC, n=10; CRC, n=10; GC, n=10; and Total, n=40). **L.** Box plots of the Kla abundance in GI cancers with different clinical stages (I, n=12; I+II, n=28). Data are presented as mean ± SEM (**J-L**). *P* values were calculated by two-sided Wilcoxon rank-sum test (**J-L**). * P < 0.05, *** P < 0.001, and **** P < 0.0001. ns indicates non-signiﬁcant.

**Figure S3**


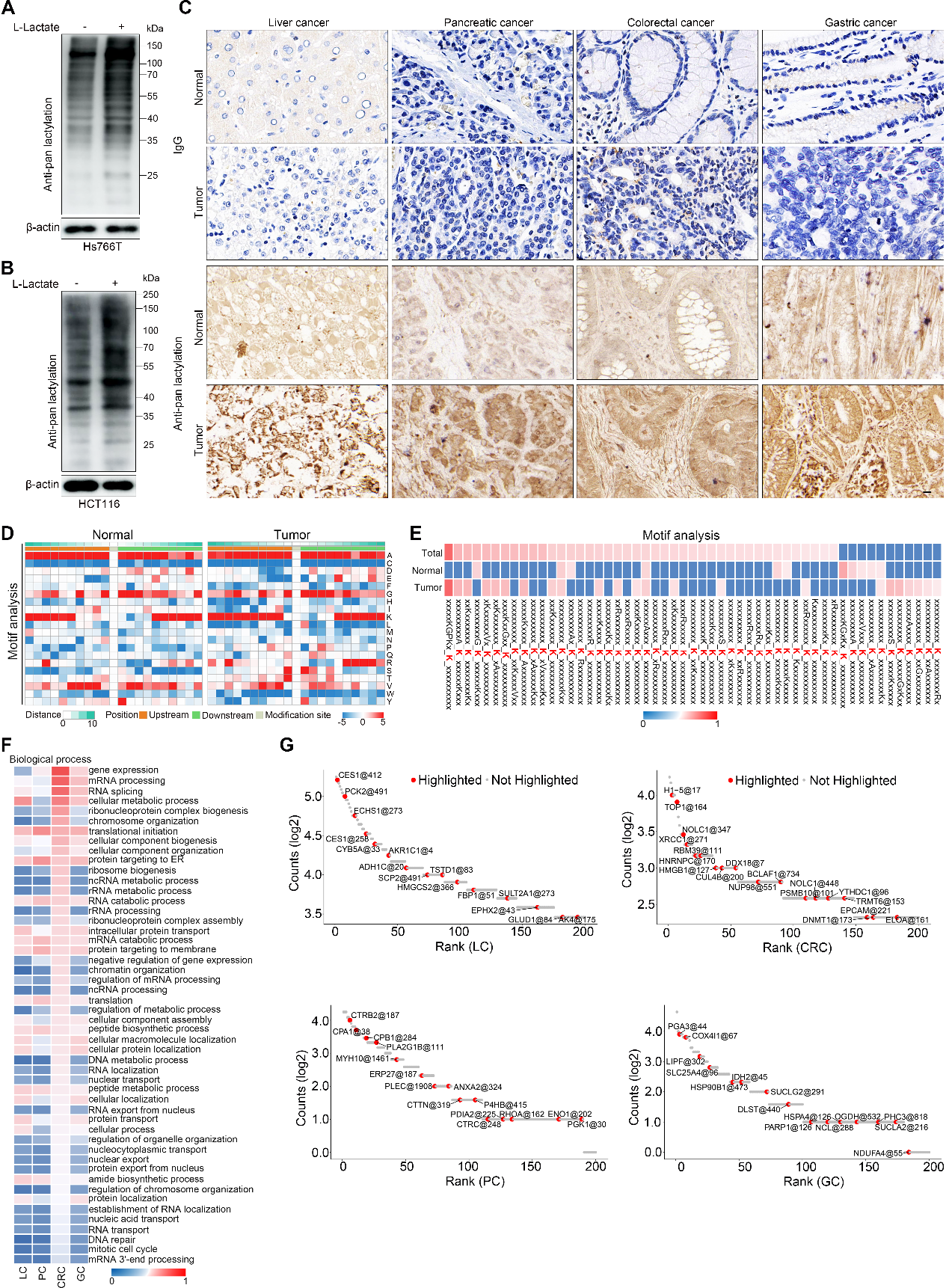


**Figure S3,** related to **Figure 1**. **Characterizing the lysine lactylation modification from patients** **with GI cancer.**

**A, B.** Hs766T and HCT116 cells were pretreated with lactate (10 mM), and total lactylation levels were determined using Western blot with anti-pan lactylation antibodies. **C**. Zoomed-out images of immunohistochemistry staining from **Figure 1F** with IgG serving as the negative control and showing the pan-Kla level in GI cancer and NATs were shown. Scale bars, 25 μm. **D.** Heatmap of the 21 amino-acid compositions of the Kla site identified from GI cancer or NATs showing the frequency of the different amino acids in specific positions flanking the lactylated lysine (red indicates greater possibility, whereas blue refers to less possibility). **E.** The ten amino acids up- and downstream of the Kla using Motif-X are analyzed and the significantly enriched motifs in indicated samples are shown. **F.** Gene Ontology biological process analysis of the enrichment of lactylated proteins in distinct GI cohorts. **G.** Relative frequencies of GI cancer-specific Kla sites ranked against the proteins.

**Figure S4**


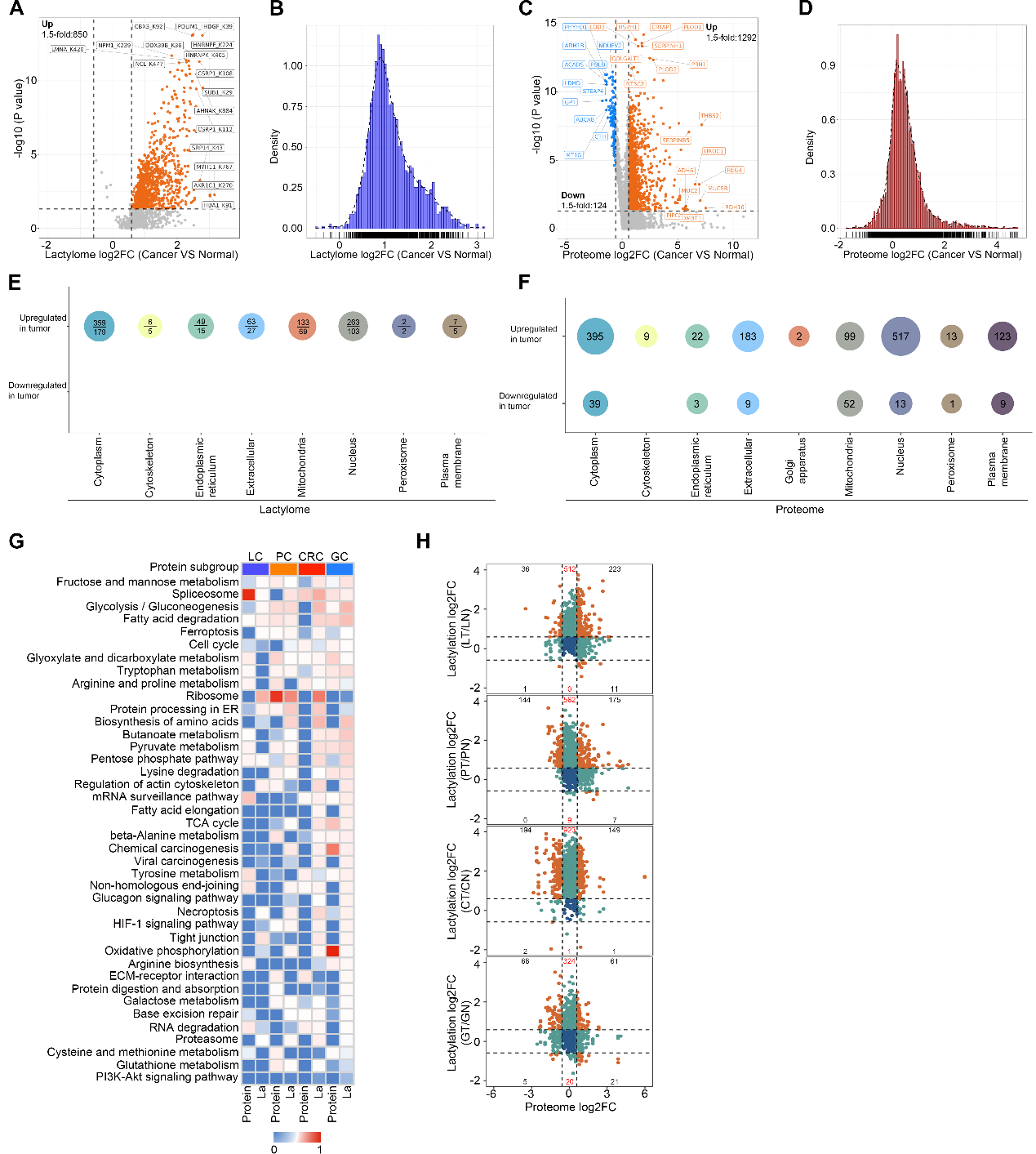


**Figure S4,** related to **Figure 2**. **Comparisons of tumor-NAT reveal GI tumorigenic changes and biomarker candidates.**

**A.** Volcano plot showing altered Kla sites in GI cancer tissues compared to those in NATs. Red and blue dots indicate upregulated and downregulated Kla sites in GI cancer tissues, respectively (n=40 samples per group). **B.** Distribution of log2-fold changes of differential Kla sites in patients. **C.** Volcano plot indicating DEPs in GI cancer tissues compared to those in NATs. Red and blue dots indicate upregulated and downregulated proteins in GI cancer, respectively (n=40 samples per group). **D.** Distribution of log2-fold changes of DEPs in patients. **E, F.** Bubble chart showing breakdown of upregulated and downregulated Kla sites (**E**) or DEPs (**F**) in GI cancer by cell compartments. **G.** Pathway enrichment analysis based on the proteins modified with differential Kla sites or the DEPs among the four GI cancer types. **H.** Scatterplots depicting fold changes of Kla compared to the protein fold changes in tumor compared to matched NAT. All identiﬁed sites are represented.

**Figure S5**

**
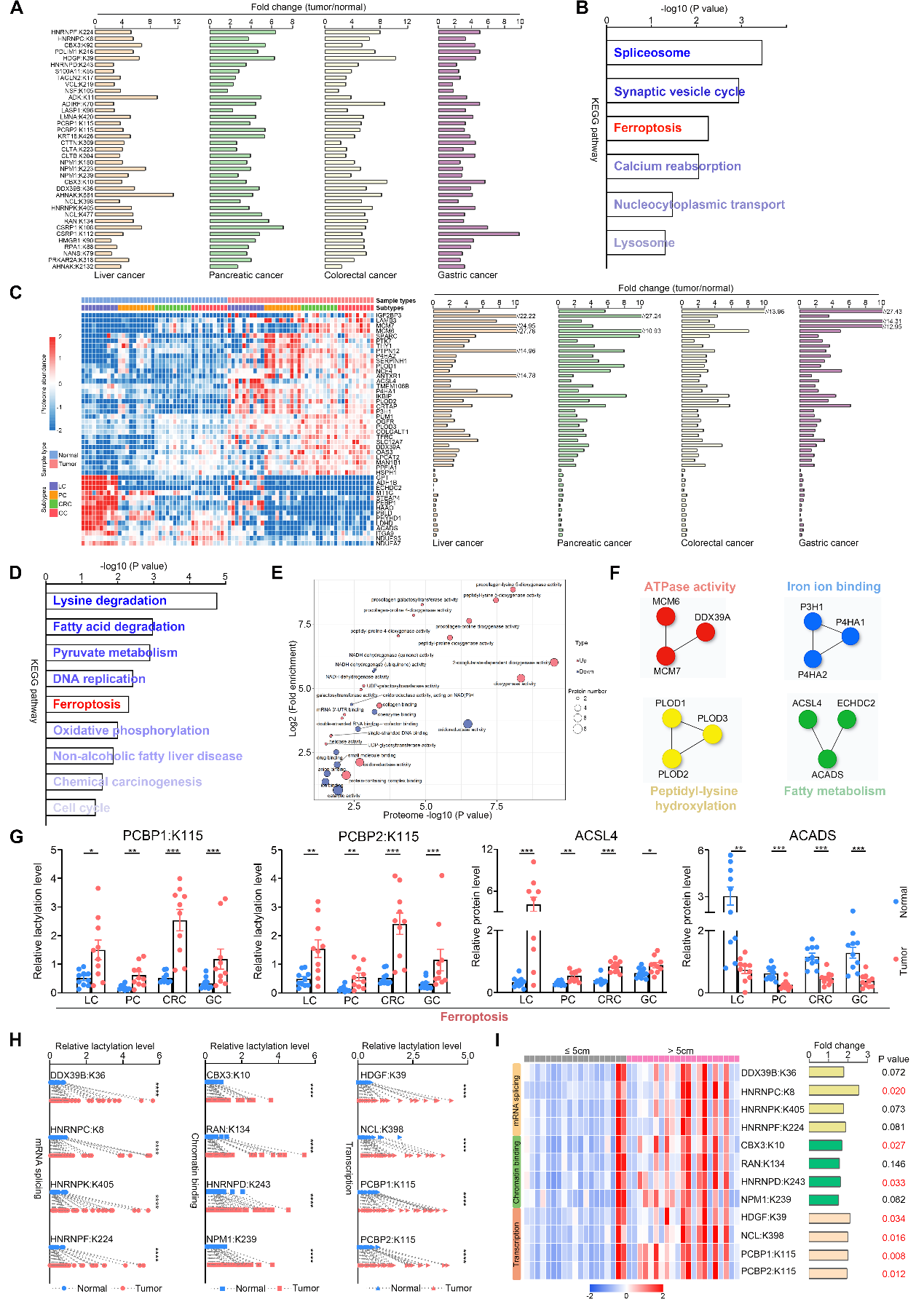
**

**Figure S5,** related to **Figure 6**. **Kla site and protein signatures targeting four types of GI cancers.**

**A.** Fold changes of the common differential 37 Kla sites in distinct GI cancers. **B.** Pathway enrichment based on common differential lactylproteins from **Figure 5A**. **C.** Hierarchical clustering of the 46 DEPs identified in all four GI tumor samples (left). Fold changes of these 46 DEPs in distinct GI cancers are showed (right). **D.** Pathway enrichment based on the 46 DEPs from **Figure S5C** (Supporting Information)**.** **E.** Gene Ontology biological processes enriched for the 46 proteins dysregulated in all four GI cancers. The X axes was plotted with -log10 (Fisher's exact test p value) and the Y axes was plotted with log2 (Fold enrichment). The node size in the graph represents the number of proteins in the specified term. **F.** Protein complex analysis of the 46 DEPs identified in all four GI cancers. **G.** Boxplots for the quantification of the indicated proteins in GI tumor and adjacent tissues (LC, n=10; PC, n=10; CRC, n=10; and GC, n=10). **H.** Differential analysis of the twelve Kla sites related to gene regulation in GI tumor compared with NATs (normal, n=40; tumor, n=40). **I.** Hierarchical clustering and fold changes of these twelve Kla sites in comparison of tumor size (≤5 cm, n=19; >5 cm, n=21). Data are presented as mean ± SEM (**G, H**). *P* values were calculated by two-sided Wilcoxon rank-sum test (**G, H**). * *P* < 0.05, ** *P* < 0.01, *** *P* < 0.001, and **** *P* < 0.0001.

**Figure S6**

**
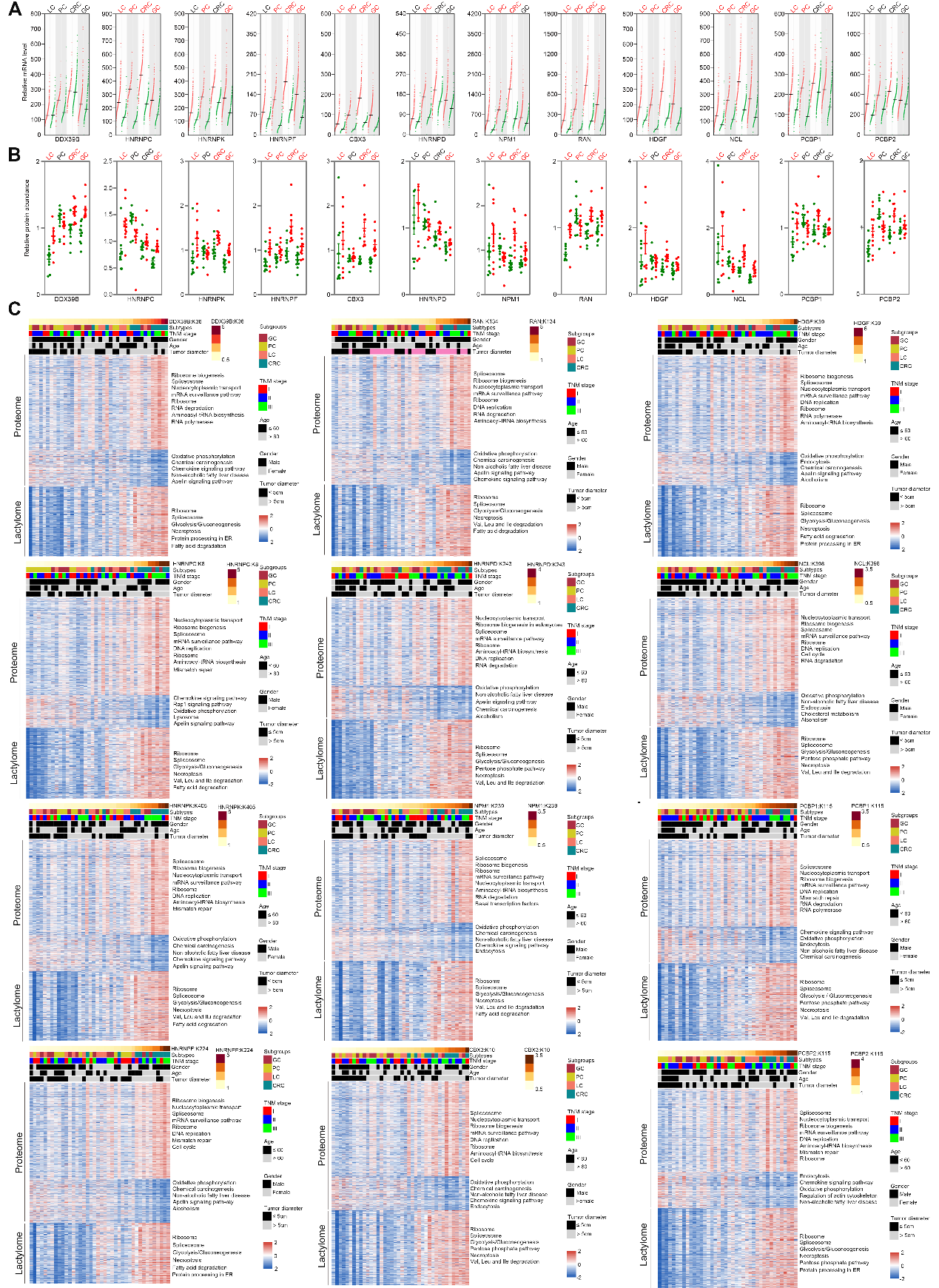
**

**Figure S6,** related to **Figure 6**. **Integrative analysis of twelve Kla sites involved in gene regulation in GI cancer.**

**A, B.** mRNA and protein expression proﬁles of the indicated genes in GI cancer and NATs. The red labels represent that the fold changes of genes were significant in the indicated GI tumors (adjusted *P* < 0.05). *P* values were calculated by two-sided Wilcoxon rank-sum test (**A, B**). **C.** Associations of indicated Kla abundance with clinicopathological characteristics and multiomics profiling.

**Figure S7**

**
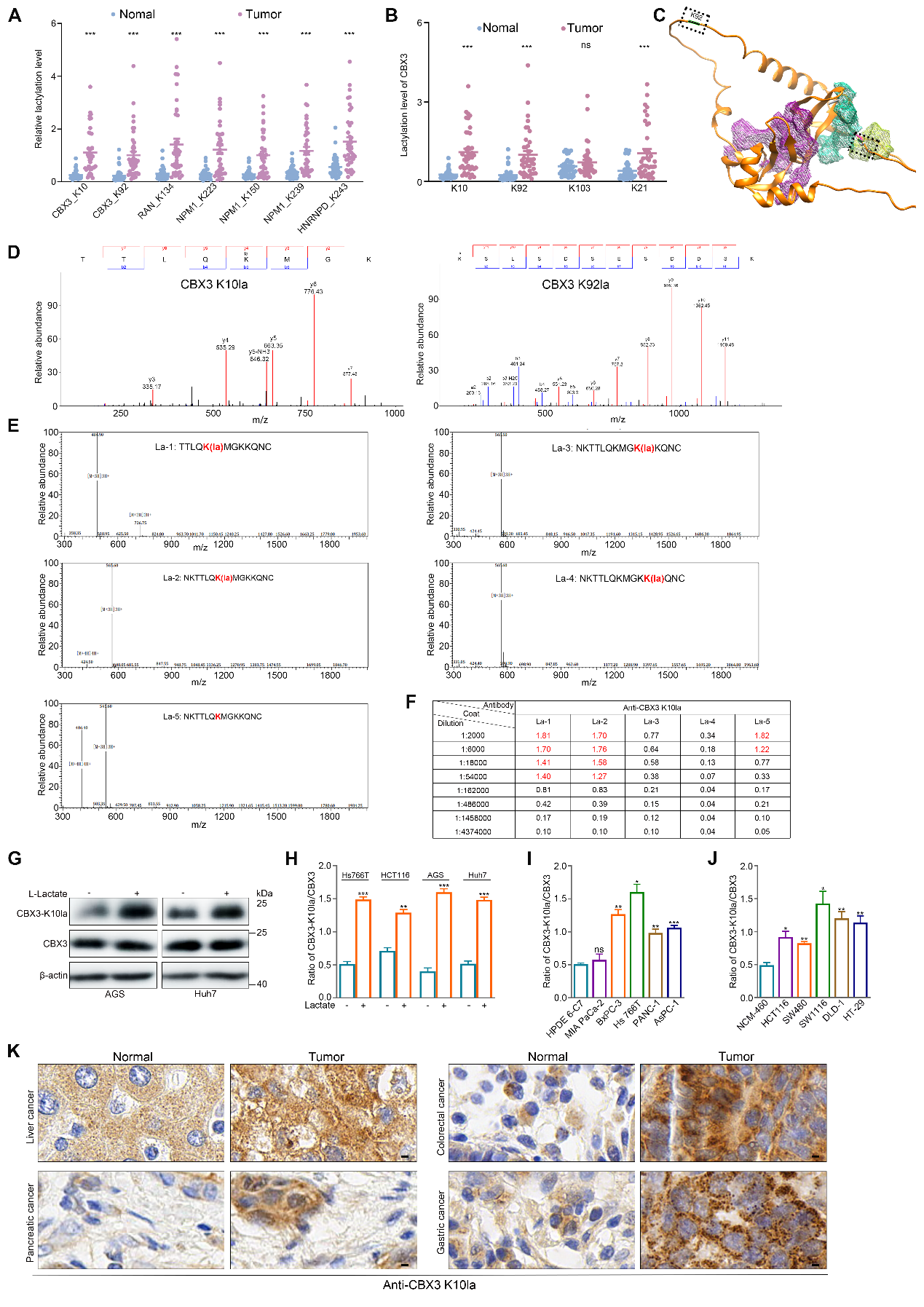
**

**Figure S7,** related to **Figure 7**. **CBX3 K10 lactylation is commonly elevated in four GI cancers.**

**A.** Differential analysis of Kla sites on proteins involved in chromatin binding in GI tumor compared with NATs (n=40 samples per group). **B.** Differential analysis of Kla sites on CBX3 in GI tumor compared with NATs (n=40 samples per group). **C.** Position analysis of K10 or K92 on CBX3 with five most enriched small molecule binding pockets. **D.** Representative spectra for peptide-containing K10 or K92 lactylation is shown. **E.** Mass spectrometry analysis of synthetic CBX3 peptides with K10 lactylation or non-lactylated at K10 for antibody preparation. **F.** Efficiency analysis of the CBX3 K10la antibody from immunity serum using routine ELISA. **G.** AGS and Huh7 cells were pretreated with lactate (10 mM), and total CBX3 and the K10la levels were determined using Western blot with anti-CBX3 and anti-CBX3-K10la antibodies. **H.** The graphs show the quantitative analysis of CBX3-K10la/CBX3 for **Figure 7F and Figure S7G** (Supporting Information) (n=3 samples per group). **I, J.** The graphs show the quantitative analysis of CBX3-K10la/CBX3 for **Figure 7G, H** (n=3 samples per group). **K.** IHC staining of the CBX3 K10la levels in GI cancer and NATs. Scale bars, 25 μm. Data are presented as mean ± SEM (**A, B,** and **H-J**). *P* values were calculated by two-sided Wilcoxon rank-sum test (**A, B**), two-tailed Welch’s t test (**H**), and one-way ANOVA with Tukey’s multiple comparison test (**I, J**). * *P* < 0.05, ** *P* < 0.01, and *** *P* < 0.001. ns indicates non-signiﬁcant.

**Figure S8**

**
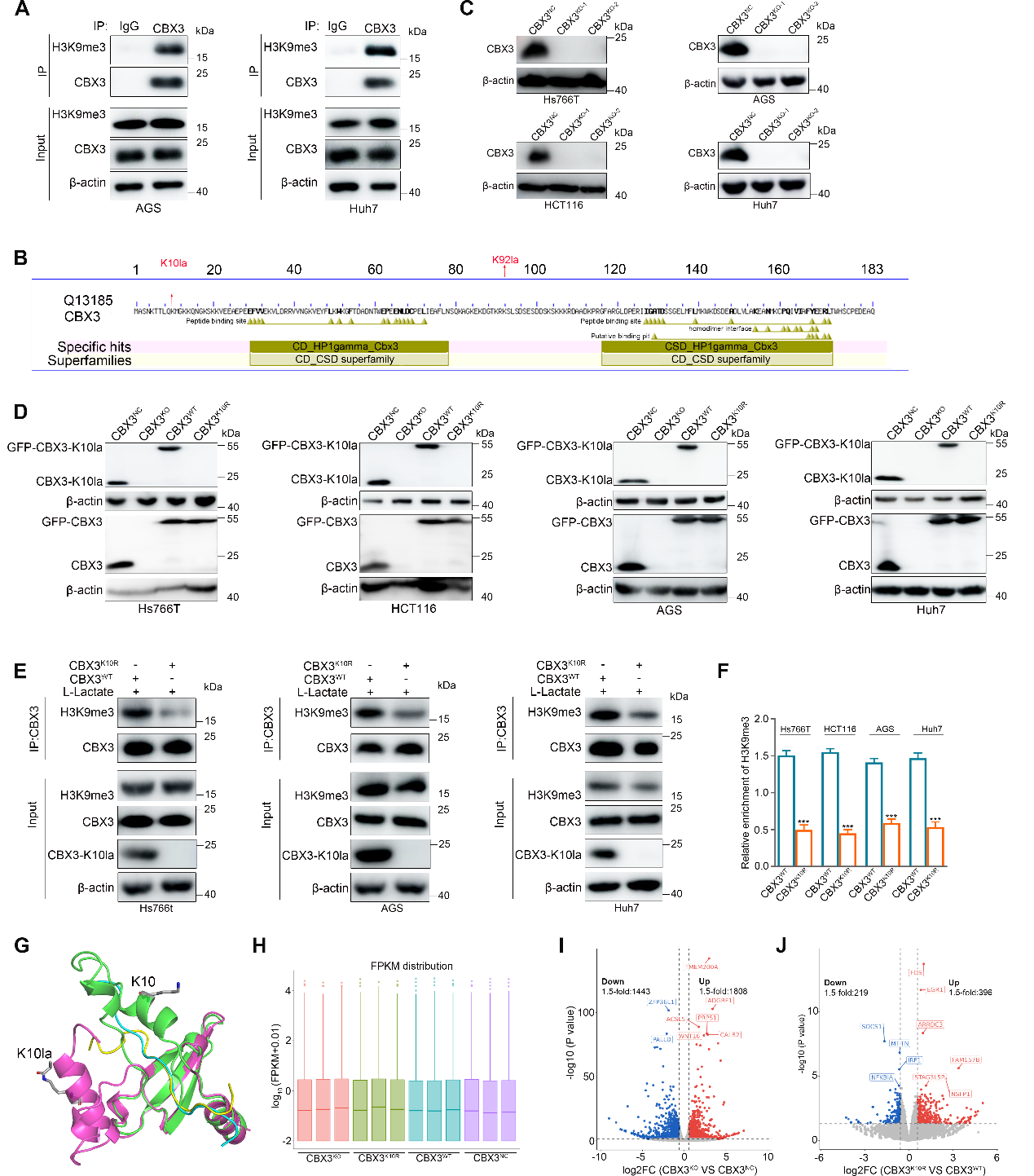
**

**Figure S8,** related to **Figure 8**. **K10 lactylation promotes the interaction of CBX3 with H3K9me3.**

**A.** Co-immunoprecipitation assay using CBX3 as bait protein with IgG as negative control demonstrated the interaction between CBX3 and H3K9me3 in AGS and Huh7 cells (n=3 biological replicates). **B.** Analysis of K10 in the domains of CBX3 based on the Conserved Domain Database. **C.** CBX3 knockout efficiency in four GI cancer cells was determined by Western blot. The sorted monoclonal cells where CBX3 has not been knocked out were defined as CBX3^NC^, and CBX3^KO^ represents CBX3 that was knocked out in indicated cells (n=3 biological replicates).  **D.** CBX3-depleted cells were transfected with lentiviral expression plasmids encoding WT CBX3 or its mutant K10R. The expression of total and lactylated CBX3 at K10 was analyzed using Western blot with anti-CBX3 and anti-CBX3-K10la antibodies (n=3 biological replicates). CBX3^WT^: CBX3-depleted cells transfected with WT CBX3; CBX3^K10R^: CBX3-depleted cells transfected with mutant CBX3 K10R. **E, F.** Co-immunoprecipitation assays were performed in CBX3^WT^ and CBX3^K10R^ cells treated with lactate (**E**). The right graphs show the quantitative analysis of relative enrichment of H3K9me3 (**F**, n=3 samples per group). **G.** Conformation changes of nonlactylated or K10-lactylated CBX3 with H3K9me3 peptide generated from molecular dynamic simulation, and K10 is shown with colored balls and sticks. **H.** The abundance of identified genes was distributed in four sets of samples that passed quality control. **I, J.** Volcano plot indicating the altered genes in CBX3^KO^ (**I**) or CBX3^K10R^ (**J**) cells. Red and blue dots indicate upregulated and downregulated genes in CBX3^KO^ or CBX3^K10R^ cells, respectively (n=3 samples per group). Data are presented as mean ± SEM (**F**). *P* values were calculated by two-tailed Welch’s t test (**F**). *** *P* < 0.001.

**Figure S9**

**
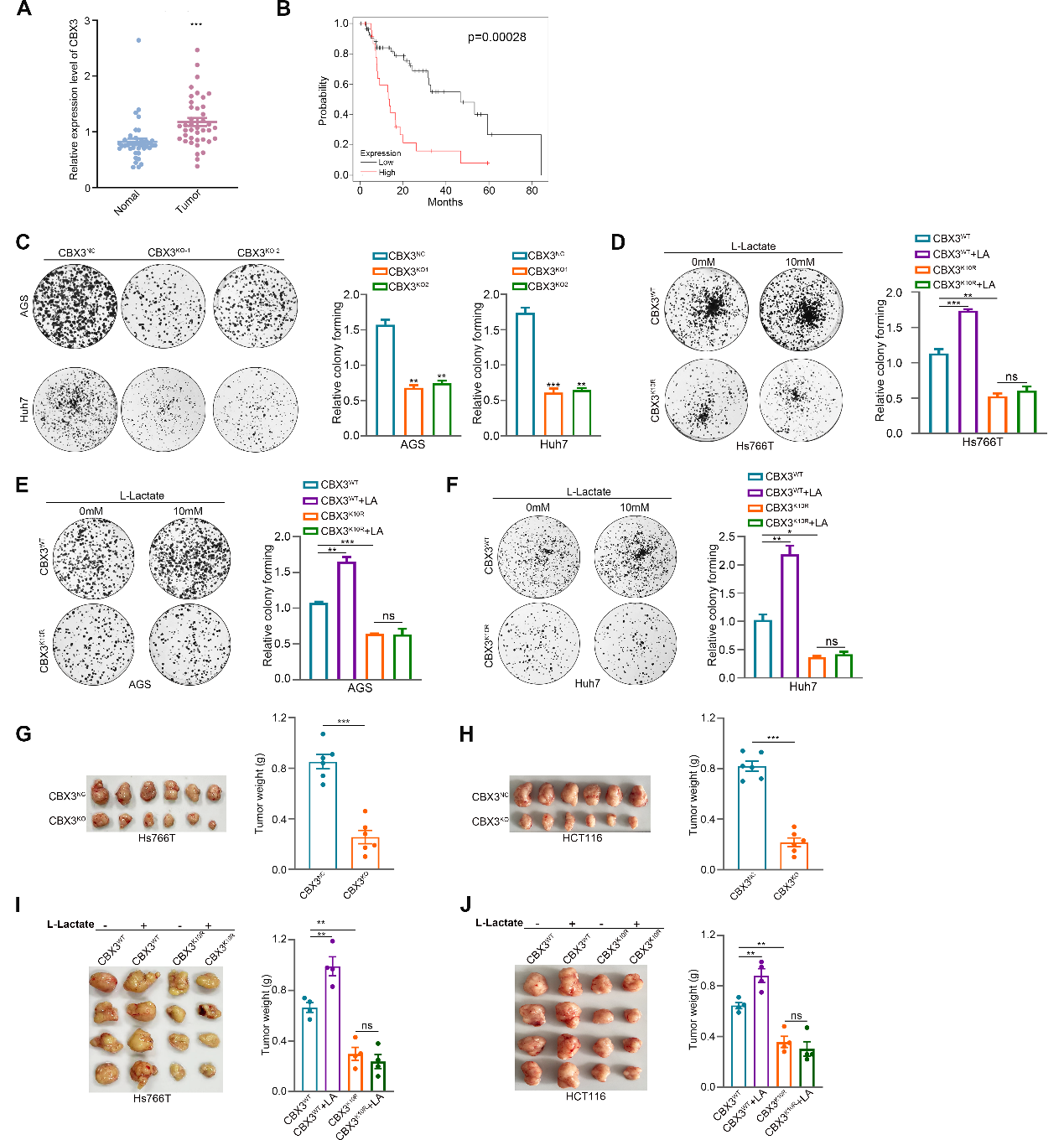
**

**Figure S9,** related to **Figure 8**. **CBX3 K10 lactylation promotes GI tumor growth.**

**A.** Protein abundance of CBX3 in GI cancer and NATs based on proteomics data (n=40 samples per group). **B.** Kaplan-Meier survival analysis of patients with pan-GI cancer using CBX3 mRNA levels in the KM Plotter database (http://kmplot.com/analysis). Statistical analysis was performed using the two-sided log-rank test. **C.** Colony formation analysis of the effect of CBX3 knockout on AGS and Huh7 cell proliferation (n=3 samples per group). **D-F.** Cell proliferation using colony formation assay in CBX3^WT^ and CBX3^K10R^ cells with treatment of lactate (n=3 samples per group). **G-J.** Nude mice were hypodermically injected with CBX3^KO^ (**G, H**, n=6 samples per group) or CBX3^K10R^ cells (**I, J**, n=4 samples per group). After approximately 1 month, tumors were dissected, photographed, and weighed. Data are presented as mean ± SEM (**A, C-J**). *P* values were calculated by two-sided Wilcoxon rank-sum test (**A**), one-way ANOVA with Tukey’s multiple comparison test (**C-F, I,** and **J**) and two-tailed Welch’s t test (**G, H**). * *P* < 0.05, ** *P* < 0.01 and *** *P* < 0.001. ns indicates non-signiﬁcant.
